# Ultrastable Conjugated Microporous Polymers Containing Benzobisthiadiazole and Pyrene Building Blocks for Energy Storage Applications

**DOI:** 10.3390/molecules27062025

**Published:** 2022-03-21

**Authors:** Mohamed Gamal Mohamed, Tharwat Hassan Mansoure, Maha Mohamed Samy, Yasuno Takashi, Ahmed A. K. Mohammed, Tansir Ahamad, Saad M. Alshehri, Jeonghun Kim, Babasaheb M. Matsagar, Kevin C.-W. Wu, Shiao-Wei Kuo

**Affiliations:** 1 Center of Crystal Research, Department of Materials and Optoelectronic Science, National Sun Yat-sen University, Kaohsiung 80424, Taiwan; mgamal.eldin12@aun.edu.eg (M.G.M.); d083100006@nsysu.edu.tw (M.M.S.); yasunou21@hotmail.co.jp (Y.T.); 2 Department of Chemistry, Faculty of Science, Assiut University, Assiut 71516, Egypt; tharout.mansour@science.au.edu.eg (T.H.M.); theoahmedkamel@gmail.com (A.A.K.M.); 3 Department of Chemistry, College of Science, King Saud University, Riyadh 11362, Saudi Arabia; tahamed@ksu.edu.sa (T.A.); alshehri@ksu.edu.sa (S.M.A.); 4 Department of Chemical and Biomolecular Engineering, Yonsei University, 50 Yonsei-ro, Seodaemun-gu, Seoul 03722, Korea; jeonghun.kim@uq.edu.au; 5 Department of Chemical Engineering, National Taiwan University, No. 1, Sec. 4, Roosevelt Road, Taipei 10617, Taiwan; matsagar03@ntu.edu.tw; 6 Department of Medicinal and Applied Chemistry, Kaohsiung Medical University, Kaohsiung 80424, Taiwan

**Keywords:** conjugated microporous polymers (CMPs), benzobisthiadiazole, thermal stability, bandgaps, energy storage

## Abstract

In recent years, conjugated microporous polymers (CMPs) have become important precursors for environmental and energy applications, compared with inorganic electrode materials, due to their ease of preparation, facile charge storage process, π-conjugated structures, relatively high thermal and chemical stability, abundance in nature, and high surface areas. Therefore, in this study, we designed and prepared new benzobisthiadiazole (BBT)-linked CMPs (BBT–CMPs) using a simple Sonogashira couplings reaction by reaction of 4,8-dibromobenzo(1,2-c;4,5-c′)bis(1,2,5)thiadiazole (BBT–Br_2_) with ethynyl derivatives of triphenylamine (TPA-T), pyrene (Py-T), and tetraphenylethene (TPE-T), respectively, to afford TPA–BBT–CMP, Py–BBT–CMP, and TPE–BBT–CMP. The chemical structure and properties of BBT–CMPs such as surface areas, pore size, surface morphologies, and thermal stability using different measurements were discussed in detail. Among the studied BBT–CMPs, we revealed that TPE–BBT–CMP displayed high degradation temperature, up to 340 °C, with high char yield and regular, aggregated sphere based on thermogravimetric analysis (TGA) and scanning electron microscopy (SEM), respectively. Furthermore, the Py–BBT–CMP as organic electrode showed an outstanding specific capacitance of 228 F g^−1^ and superior capacitance stability of 93.2% (over 2000 cycles). Based on theoretical results, an important role of BBT–CMPs, due to their electronic structure, was revealed to be enhancing the charge storage. Furthermore, all three CMP polymers featured a high conjugation system, leading to improved electron conduction and small bandgaps.

## 1. Introduction

The news that the depletion of natural sources of energy has compelled researchers to search for alternatives that provide high energy and power density, long cycle life, good reversibility, and good sources of energy storage is not new. Among many other energy storage systems (ESSs), the focus on supercapacitors has increased due to their high stability as a function of temperature, excellent cyclability, easy fabrication, high energy, and power density [1,2,3,4,5,6,7]. Supercapacitors store charge via two mechanisms—electrostatically and faradaically [1,7]. The electrostatic mechanism stores the charges at the interface of the supercapacitor electrode as electric double layer capacitance (EDLC), while the faradaic mechanism stores the charges at the electrode surface as pseudocapacitance (PC) due to the highly reversible surface redox reaction of the redox-active sites during charge and discharge [1,7]. The ELDC of the materials can be attributed to their surface areas. The materials that have high surface areas, such as carbonaceous materials, conjugated microporous polymers (CMPs), and covalent organic frameworks (COFs), usually have high EDLC [8,9,10,11,12,13,14,15]. It is, therefore, important to explore new electrode materials that synergize both EDLC and PC. With this in consideration, an ideal electrode material should meet several characteristics, such as having ideal redox-active sites, high specific surface area (SSA), good conductivity, suitable pore width, and good chemical and thermal stability, as well as being eco-friendly in nature. Extensive research on CMPs reveals that the introduction of heteroatoms (N, B, S, P) in the CMPs can be used to obtain materials with tunable pore size and PC [16,17,18,19,20,21].

In recent years, researchers across the globe have been dedicated—at both academic and industrial levels—to enhance the use of renewable and sustainable energy sources in order to limit the impact of fossil foil resources on the environment [22]. Most commonly, polymers in polymeric electrolytes are categorized into synthetic and natural types; synthetic polymers such as polyethylene, polyvinyl chloride, polymethyl methacrylate, polycarbonate, and polyacrylonitrile are nondegradable and are of serious concern for environmental well-being [23]. Additionally, considerable progress has been achieved in the production of energy from renewable sources during the preceding two decades, which has triggered the need for both reliable and powerful energy resources while also being environmentally benign [24]. Recently, various biodegradable polymers such as cellulose, carbohydrates, and chitosan with appropriate modification to enhance their ionic conductivity have been employed as electrolytes in different energy storage devices such as batteries, dye-sensitized solar cells, and supercapacitors [25].

CMPs are porous compounds that are constructed from a covalent connection of π-conjugated building units that extended along with the skeleton, providing them 3D microporosity networks and especially high surface area at times [26,27,28,29,30,31,32,33,34]. They can be easily synthesized by using numerous C–C coupling reactions such as Yamamoto, Sonogashira, and Suzuki coupling, and also oxidative polymerization [35,36,37,38,39,40,41,42,43,44] which are characterized by a high degree of polymerization and crosslinking densities, as well as facilities the incorporation of different moieties [45,46,47,48,49,50]. These coupling methods allow CMPs to highly possess CMPs to differentiate from other conventional polymers, with rapid ion/diffusion kinetics that originates from their individual porous properties [51,52,53,54,55]. Therefore, CMPs displayed high capacity and long-term cycling stability when they were applied as material electrodes in electrochemical energy storage applications [56,57,58,59,60]. Hence, they are considered as one of the ideal candidates for catalysis, gas storage, and electrochemical energy storage [61,62,63,64].

In recent years, benzobisthiadiazole moiety (BBT) is considered a strong withdrawing electron group and a promising molecule, which has widely been applied in organic photovoltaics, nonlinear optics, organic field-effect transistor, and organic light-emitting diodes, due to its charge carrier mobilities and low bandgap value [65,66,67,68]. This study is the first report to prepare CMP materials containing benzobisthiadiazole units and use these materials as an organic electrodes for supercapacitor application. Herein, we successfully constructed new BBT–CMPs containing benzobisthiadiazole, triphenylamine (TPA), pyrene (Py), and tetraphenylethene (TPE) moieties through Sonogashira coupling’s reaction, and their structure and properties such as porosity, thermal stability, and surface properties were analyzed using solid-state NMR, scanning electron microscopy (SEM), transmission electron microscopy (TEM), and N_2_ adsoprtion measurements. According to electrochemical data, our three new BBT–CMPs showed outstanding capacitance response of up to 210 F g^−1^ with superior retention of capacity over long 2000 cycles. These results were associated with the theoretical studies, which were carefully carried out by using density functional theory (DFT), to calculate the distribution of different energy gaps such as the highest occupied molecular orbital (HOMO) and the lowest unoccupied molecular orbital (LUMO), and molecular electrostatic potential (MESP), respectively. Interestingly, the obtained theoretical results confirmed that the electronic structure of the BBT–CMP plays a critical role to improve charge storage. Furthermore, this study will pave the way for other applications such as hydrogen evolution and lithium batteries using BBT–CMPs due to their extended π-conjugation leading, small bandgaps, and highly delocalized LUMO distribution.

## 2. Results

### 2.1. Synthesis and Character of BBT Monomers and BBT–CMPs

**Scheme 1:**
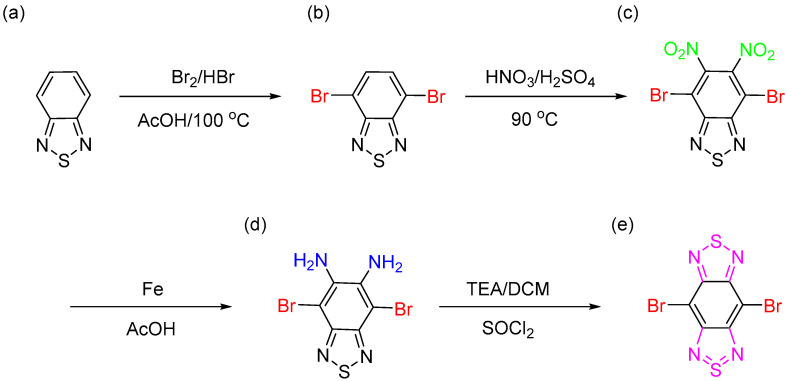
Synthetic method of BT–Br_2_ (**b**), BT–2NO_2_ (**c**), BT–2NH_2_ (**d**) and BBT–Br_2_ (**e**) preparation from BT (**a**).

**Scheme 2:**
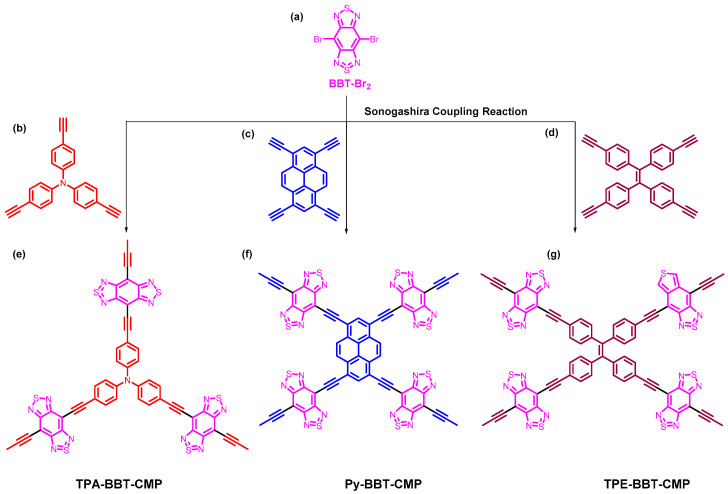
Synthetic scheme of the TPA–BBT–CMP (**e**), Py–BBT–CMP (**f**) and TPE–BBT–CMP (**g**) from BBT–Br_2_ (**a**), TPA-T (**b**), Py-T (**c**), and TPE-T (**d**).

BBT–Br_2_ monomer was used as a building unit to prepare the different types of CMPs in this study for energy storage application, as displayed in Scheme 1. First, bromination reaction of 2,1,3-benzothiadiazole moiety (BT) (Scheme 1a) in the presence of aqueous solution HBr in acetic acid and Br_2_ solution was made to afford BT–Br_2_ as a white crystalline powder with a high yield of up to 85% (Scheme 1b). Then, we carried out the nitration reaction of BT–Br_2_ by using both concentrated HNO_3_ and H_2_SO_4_ to obtain BT–2NO_2_, as displayed in Scheme 1c. The formation of BBT–Br_2_ was successfully prepared through two steps by the reduction reaction of BT–2NO_2_ in the presence of Fe powder in acetic acid solution to afford BT–2NH_2_ (Scheme 1d), followed by ring closure reaction by using SOCl_2_ in a mixture of triethylamine (TEA) and dichloromethane (DCM) to yield BBT–Br_2_ (Scheme 1e) as a reddish-brown solid. The chemical formula of all of the synthesized monomers were confirmed by using Fourier transform infrared spectroscopy (FTIR), proton nuclear magnetic resonance (^1^H-NMR), and mass spectroscopy analyses. The synthetic method for the synthesis of three novel CMPs based on the BBT unit is shown in Scheme 2e–g. The reaction of BBT–Br_2_ (Scheme 2a) as the main building monomer with three different monomers containing alkynyl groups such as TPA-T (Scheme 2b), Py-T (Scheme 2c), and TPE-T (Scheme 2d) by using Sonogashira coupling reaction in the presence of Pd as a catalyst in DMF–TEA mixture (1:1) for 72 h to afford of TPA–BBT–CMP as a brown solid, Py–BBT–CMP as a reddish-brown powder, and TPE–BBT–CMP as an orange solid, respectively (Scheme 2e–g).

**Figure 1:**
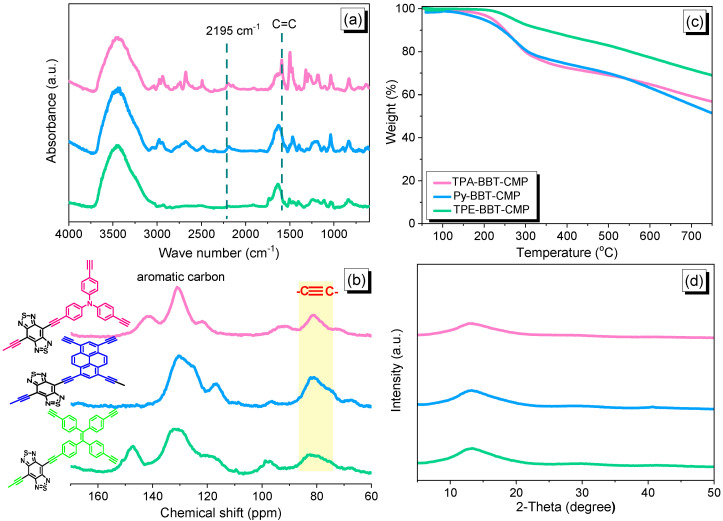
(**a**) FTIR, (**b**) TGA, (**c**) solid-state NMR, and (**d**) XRD spectra of the TPA–BBT–CMP, Py–BBT–CMP, and TPE–BBT–CMP.

Figure 1a represents the FTIR spectra of TPA–BBT–CMP, Py–BBT–CMP, and TPE–BBT–CMP, respectively. As observed in Figure 1a, all three BBT–CMP samples appeared corresponding absorption bands in the range of 3400, 2198, and 1624 cm^−1^, corresponding to the stretching of OH group that belong to water absorption by CMP materials and stretching vibration of terminal C≡C and C=C groups, respectively. In addition, all BBT–CMPs featured absorption signals centered near 3045–3035 cm^−1^ due to vibrations of the stretching aromatic C–H. The presence of aromatic carbon nuclei and internal C≡C signals in the three BBT–CMPs appeared in the range 116–146 ppm and 81 and 78 ppm as presented in ^13^C solid-state NMR (Figure 1b). In addition, the carbon nuclei of C=C units in the TPE moiety in the TPE–BBT–CMP spectrum were found to be 142 ppm. We evaluated the thermal stability of TPA–BBT–CMP, Py–BBT–CMP, TPE–BBT–CMP, and their corresponding monomers via TGA under the N_2_ atmosphere from 100 to 800 °C (Figure 1c, Figures S7, S11 and S15, Table 1 and Table S1). The thermal decomposition temperatures at 10% weight loss (T_d10_) and char yields were 256 °C and 15 wt% for BT–2NO_2_; 228 °C and 0 wt% for BT–2NH_2_; 262 °C and 13 wt% for BBT–Br_2_. As shown in TGA profiles in Figure 1c, the weights of TPA–BBT–CMP, Py–BBT–CMP, and TPE–BBT–CMP remained constant up to 250 °C, with subsequent 10 wt% weight losses at 250, 244, and 347 °C, respectively; their char yields at 800 °C were 55, 48, and 67 wt%, respectively (Figure 1c). The powder X-ray diffraction (PXRD) measurements of all BBT–CMP samples exhibited amorphous character without possessing any ordered diffraction peaks, as illustrated in Figure 1d.

### 2.2. Porosity and Morphologies Properties of BBT–CMPs

**Figure 2:**
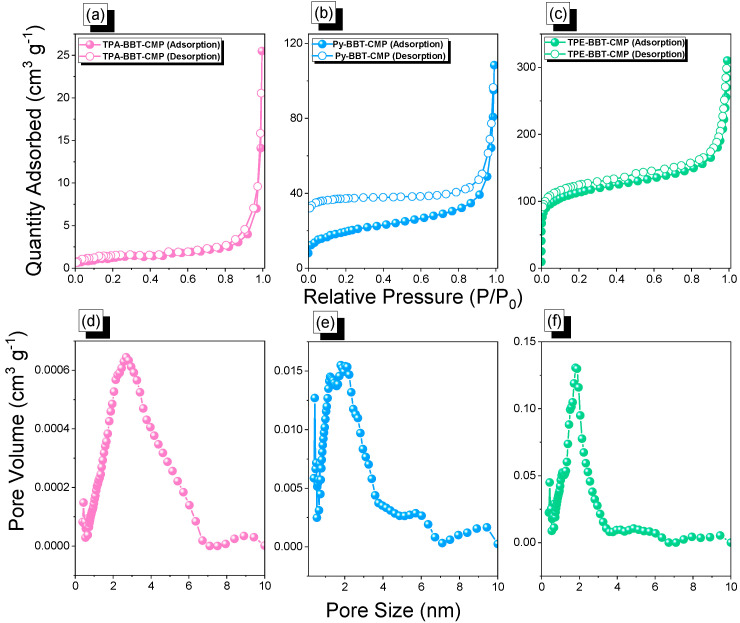
N_2_ Uptake and pore size diameter curves of the TPA–BBT–CMP (**a**,**d**), Py–BBT–CMP (**b**,**e**), and TPE–BBT–CMP (**c**,**f**).

N_2_ adsorption–desorption tests were performed at 77 K and 1 bar to corroborate the porosity characteristics of the TPA–BBT–CMP, Py–BBT–CMP, and TPE–BBT–CMP (Figure 2a–c and Table 1). Based on IUPAC classification, the N_2_ uptake isotherms were type III for TPA–BBT–CMP, type II for Py–BBT–CMP, and type II and IV for TPE–BBT–CMP. Both N_2_ profiles of the Py–BBT–CMP and TPE–BBT–CMP showed rapid N_2_ uptake at a low and high relative pressure (P/P_0_), indicating the presence of micro- and mesoporosity properties within these two materials. The specific surface area and total pore volumes of TPA–BBT–CMP, Py–BBT–CMP, and TPE–BBT–CMP were found to be 35.6 m^2^ g^−1^ and 0.16 cm^3^ g^−1^, 67 m^2^ g^−1^ and 0.024 cm^3^ g^−1^, and 410 m^2^ g^−1^ and 0.50 cm^3^ g^−1^, respectively. Finally, the pore size diameters of TPA–BBT–CMP, Py–BBT–CMP, and TPE–BBT–CMP were 2.7, 1.3, and 1.8 nm, respectively (based on nonlocal density functional theory (NL-DFT)) (Figure 2d–f). 

**Figure 3:**
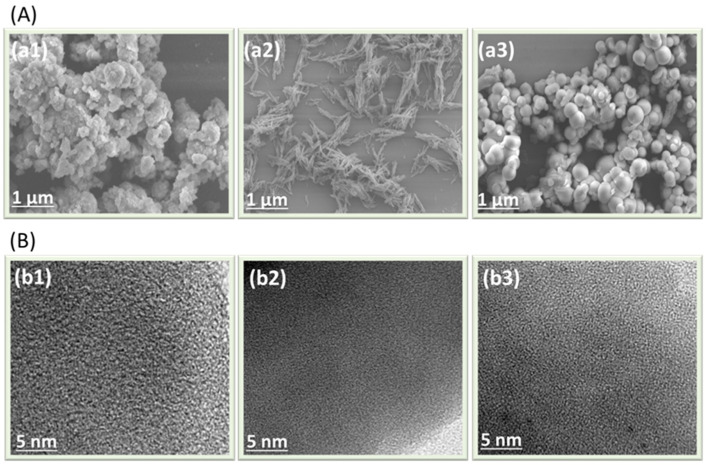
(**A**) SEM and (**B**) TEM images of the TPA–BBT–CMP (**a1**,**b1**), Py–BBT–CMP (**a2**,**b2**), and TPE–BBT–CMP (**a3**,**b3**).

The surface morphological of TPA–BBT–CMP, Py–BBT–CMP, and TPE–BBT–CMP featured irregular aggregated sphere, rod, and regular sphere aggregate structures, respectively, as displayed in SEM images (Figure 3A(a1–a3)). Furthermore, TEM images of all BBT–CMPs are shown in Figure 3B(b1–b3), which revealed the presence of small pores and the presence of dark and bright areas in their structure, confirming their non-order and amorphous nature.

### 2.3. Electrochemical Performance of BBT–CMPs

**Figure 4:**
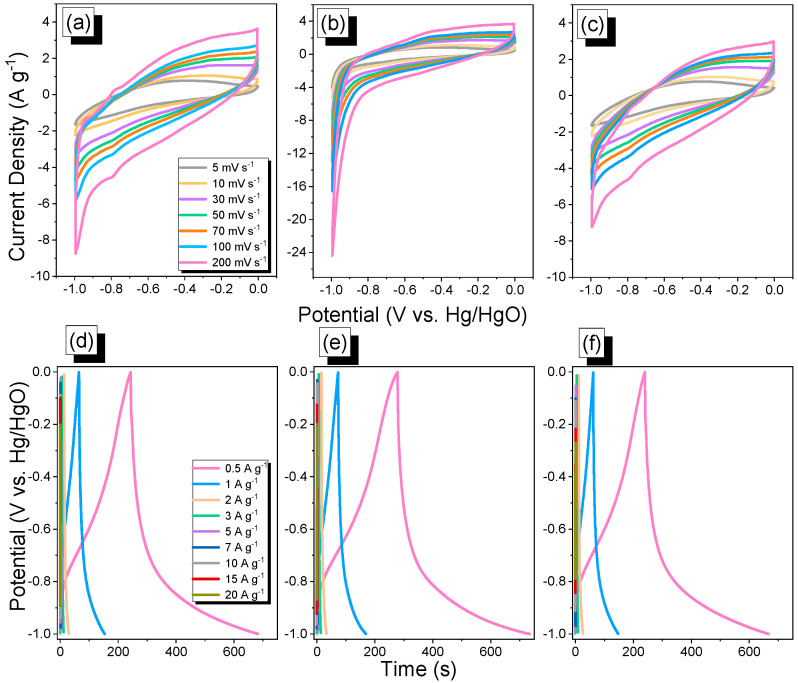
CV and GCD analyses of the TPA–BBT–CMP (**a**,**d**), Py–BBT–CMP (**b**,**e**), and TPE–BBT–CMP (**c**,**f**).

The electrochemical performance of BBT–CMP samples was evaluated using cyclic voltammetry (CV) and galvanostatic charge–discharge (GCD) analyses using glassy carbon (as the working electrode), Hg/HgO (as reference electrode), and platinum wire (as a counter electrode) in 1 M KOH aqueous solution. Figure 4a–c display the corresponding CV curves of BBT–CMP samples at different scan rates from 5 to 200 mV s^−1^, recorded within the potential window of 0.00 to −1.00 V (vs. Hg/HgO). The synergism of ELDC and PC can be confirmed from the rectangle-shaped, humped CV curves of all the BBT–CMPs, even at a high scan rate of 200 mV s^−1^ [2,12,19]. The CV curves of all the BBT–CMPs exhibit a little deviation from the capacitive nature due to the pseudocapacitive effect [69,70], which is the result of fast redox reactions occurring on and near the surface of active materials during charge and discharge [69,71]. As shown in Figure 4a–c, the presence of a hump/spike indicates the contribution of pseudocapacitance, which arose from the presence of various types of nitrogen and sulfur heteroatoms that enable easier accessibility of electrolytes to the electrode surface and transfer of electrons [72,73]. The Py–BBT–CMP sample showed a higher integrated area than the other two samples (TPA– and TPE–BBT–CMP), which reveals its superior electrochemical performance. Furthermore, increasing the sweep rate from 5 to 200 mV s^−1^ enhanced the current density while maintaining the form of the CV curves, suggesting strong rate capability and simple kinetics. Figure 4d–f represent the GCD curves of the BBT–CMP samples, recorded within the potential window of 0.00 to −1.00 V (vs. Hg/HgO) at various current densities from 0.5 to 20 A g^−1^. The GCD curves of all BBT–CMP samples displayed triangular forms with a small bend, suggesting that the presence of distinct kinds of N and S heteroatoms caused both EDLC features and pseudocapacitance [2,12,14]. The discharging times of the Py–BBT–CMP (455.6 sec at 0.5 A g^−1^) sample was longer than that of both TPA–BBT–CMP (439 s at 0.5 A g^−1^) and TPE–BBT–CMP (426.6 s at 0.5 A g^−1^) samples (Figure 4d–f).

**Figure 5:**
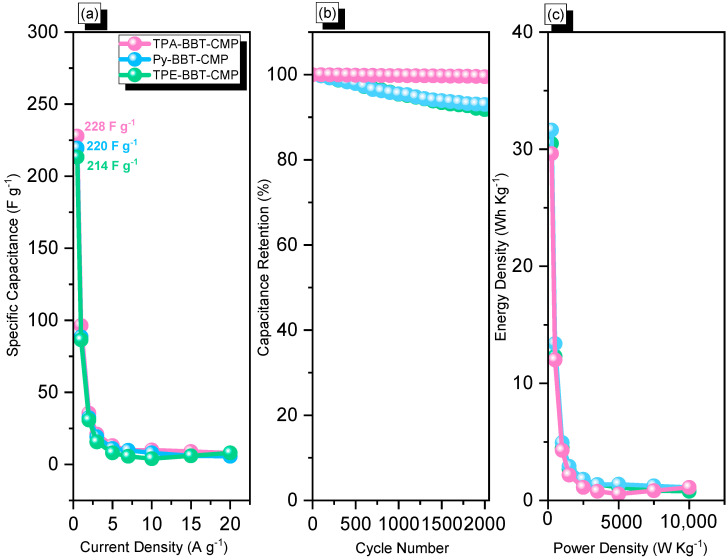
Capacitance (**a**), stability (**b**), and Ragone plots (**c**) of TPA–BBT–CMP, Py–BBT–CMP, and TPE–BBT–CMP.

Figure 5a shows the specific capacitances of all BBT–CMP samples determined from GCD curves using Equation (1).

*C*_s_ = (*I*Δ*t*)/(*m*Δ*V*) (1)

where *C*_s_ (F g^−1^) is the specific capacitance of the supercapacitor, *I* (A) is the discharge current, Δ*V* (V) is the potential window, Δ*t* (s) is the discharge time, and *m* (g) is the mass of the NPC on the electrode.

All BBT–CMP samples displayed superior capacitance (TPA–BBT–CMP, 220 F g^−1^; Py–BBT–CMP, 228 F g^−1^; TPE–BBT–CMP, 214 F g^−1^, at 0.5 A g^−1^), compared with other porous materials (Table S2). This behavior could be explained by the presence of various types of N and S heteroatoms, which enable easier accessibility of electrolytes to the electrode surface. In addition, upon increasing the current density from 0.5 to 20 A g^−1^, the specific capacitances of all BBT–CMP samples decreased due to the insufficient time available for ion diffusion at high current densities. Our lab has recently made two ferrocene-derived conjugated microporous polymers (FFC–CMPs) via Sonogashira couplings [12], and these materials showed low values of specific capacitances at 0.5 A g^−1^ (Table S2). In addition, we prepared three CMPs (TBN–TPE–CMP, TBN–Py–CMP, TBN–Car–CMP) via Sonogashira–Hagihara cross-couplings [19]. The electrochemical performance of the three TBN–CMP samples revealed specific capacitances in the range 18.45, 31, and 18.90 F g^−1^. Moreover, we prepared two-hybrid porous polymers (POIPs) through the Heck reaction of octavinylsilsesquioxane with brominated fluorene and anthraquinone (A-Br_2_) with octavinylsilsesquioxane (OVS) [10]. We revealed that POSS (polyhedral oligomeric silsesquioxane)-A-POIP had higher capacitance than POSS-F-POIP due to the π-conjugated system and faradaic reaction of the anthraquinone unit. The durability of all samples was tested through GCD measurements over 2000 cycles at a current density of 10 A g^−1^ (Figure 5b). The TPA–BBT–CMP, Py–BBT–CMP, and TPE–BBT–CMP samples displayed good cycling stability (Figure 5b and Figure S18), with 91.8%, 93.2%, and 99.5% retention, respectively, of their original capacitances after 2000 cycles, indicating their excellent cycling stability in 1 M KOH as the electrolyte. Energy (*E*, W h kg^−1^) and power densities (*P*, W kg^−1^) were calculated using Equations (2) and (3), respectively, to build the Ragone plot.

*E* = 1000*C*(Δ*V*)^2^/(2 × 3600) (2)

*P* = *E*/(*t*/3600) (3)

The Ragone plot (Figure 5c) showed that the energy and power densities of the Py–BBT–CMP sample were higher than those of the TPA–BBT–CMP and TPE–BBT–CMP samples, suggesting that the Py–BBT–CMP sample possessed good energy and power densities.

**Figure 6:**
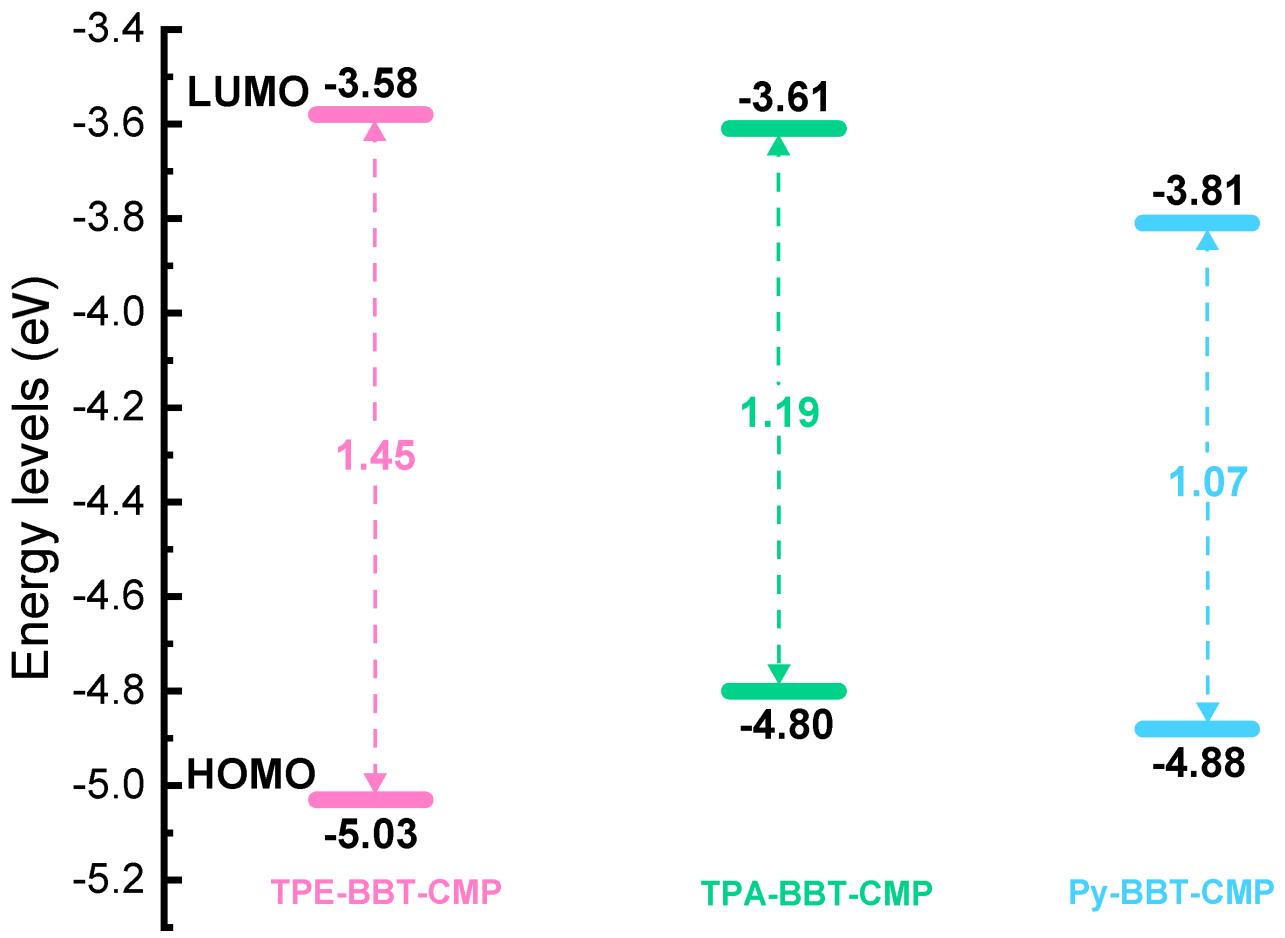
HOMO–LUMO diagrams for TPE–BBT–CMP, TPA–BBT–CMP, and Py–BBT–CMP.

We investigated the electronic structure of the monomers and polymers using density functional theory (DFT) to gain a better understanding of the three BBT–CMP polymers’ superior electrochemical performance. The charge storage ability and redox characteristics of CMPs are influenced by their electronic structure. The basis set 6–31G was employed using the hybrid functional B3LYP (d). To accommodate for long-range and noncovalent interactions, we employed the D3BJ dispersion correction. For the ground state geometry of the monomers, we investigated many conformers and chose the one with the lowest energy. The global minimum was then confirmed using harmonic vibrational frequency. At the same level of theory, the highest occupied molecular orbital (HOMO) and lowest unoccupied molecular orbital (LUMO) energy gaps, as well as the molecular electrostatic potential (MESP), were calculated on the optimized geometries. The LUMO distribution has a significant influence on the polymer’s electrochemical performance. The high delocalization degree of LUMO orbitals endows the BBT–CMP polymers with high n-doping activity. Moreover, the narrow bandgap of the BBT–CMP polymers (Figure 6) leads to a higher electron conductivity and, as a result, superior electrochemical performance.

**Figure 7:**
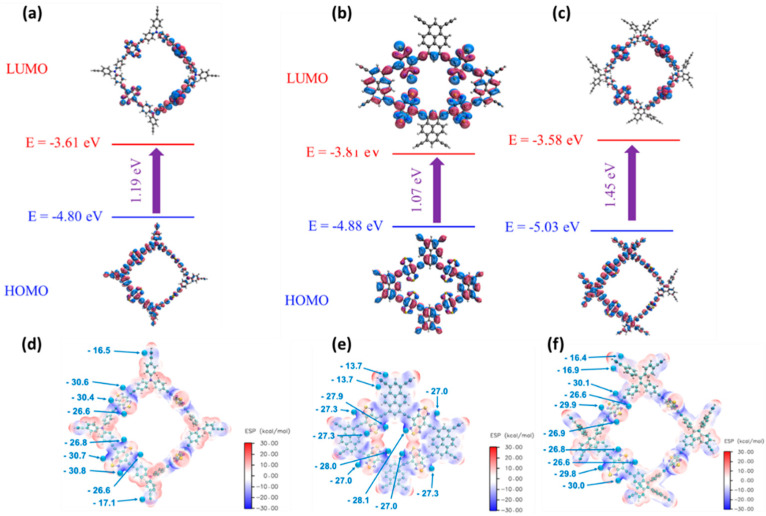
Energy levels and MESP diagrams of the TPA–BBT–CMP (**a**,**d**), Py–BBT–CMP (**b**,**e**), and TPE–BBT–CMP (**c**,**f**).

Figure 7 and Figure S19 presented the frontier molecular orbitals of the building blocks monomers (BBT, TPE, TPA, Py, and TPE) and the synthesized three BBT–CMPs. Both HOMO and LUMO are highly delocalized and are spread all over the whole molecule for the building blocks monomers and BBT–CMP polymers, as shown in Figure 7a–c and Figure S19, respectively. Figure 7d–f depict the molecular electrostatic potential (MESP) analysis of TPE–BBT–CMP, TPA–BBT–CMP, and Py–BBT–CMP samples. Figure S20 presents the molecular electrostatic potential (MESP) analysis of the building blocks monomers (TPE, TPA, Py, and BBT). The high delocalization leads to decreasing the charge density over active sites. Low negative charge density at redox-active sites (N in all polymers) may impair their contact with the electrolyte, allowing for quicker ion diffusion and electron transfer through CMP pores. The prolonged conjugation in all three polymers leads to improved electron conduction, a highly delocalized LUMO distribution, tiny band gaps, and lower negative charge density on redox-active sites. These characteristics boost the electrochemical performance of these polymers.

## 3. Synthesis Methods

### 3.1. Materials

*N*,*N*-dimethylformamide (DMF, 99.9%), thionyl chloride (SOCl_2_, 99%), bromine solution (Br_2_, 99.5%), triphenylamine (TPA, 98%), hydrobromic acid (HBr, 48%), benzophenone (99%), triethylamine (Et_3_N, 99.5%), dichloromethane (DCM), methanol (MeOH), tetrahydrofuran (THF), acetone, hydrochloric acid solution (HCl, 12 M), Pd(PPh_3_)_4_ (99%), and sodium carbonate (NaCO_3_, 99.5%) were purchased commercially from Alfa Aesar (Lancashire, UK), Acros (Fukuoka, Japan), and Sigma–Aldrich (Burlington, MA, USA). 1,3,6,8-tetraethynylpyrene (Py-T), and 1,1,2,2-tetrakis(4-ethynylphenyl)ethene (TPE-T) were synthesized according to previously reported procedures [19,41].

### 3.2. Synthesis of 4,7-Dibromobenzo[c][1,2,5]thiadiazole (BT-Br_2_)

BT (10.0 g, 73.4 mmol), 80 mL of HBr (48%), and (35.2 g, 220.3 mmol) of Br_2_ solution were mixed, and the mixture was refluxed at 95 °C for 9 h. Then, the mixture solution was added into NaOH solution at 0 °C, and the total solution was worked up by using DCM to afford a BT–Br_2_ as a white powder (5 g, 50%). FTIR (Figure S1): 3035. ^1^H NMR (Figure S2): 7.73 ppm. ^13^C NMR (Figure S3): 153.5, 133, 114.50 ppm.

### 3.3. Synthesis of 4,7-Dibromo-5,6-dinitrobenzo[c][1,2,5]thiadiazole (BT–2NO_2_)

BT–2NO_2_ was synthesized via the nitration of BT–Br_2_ (2 g, 6.8 mmol) with 28 mL of conc. H_2_SO_4_ and 14 mL of fuming HNO_3_ at 0 °C, and the solution was refluxed up to 90 °C for 24 h. Then, the mixture solution was added into Na_2_CO_3_ solution at 0 °C to provide a white crystal (1.30 g, 65%). FTIR (Figure S4): 1343 cm^−1^ (NO_2_ group). ^1^H NMR (Figure S5): no signal observed. (+) ESI-MS (Figure S6): 381.10. T_d10_ = 256 °C (Figure S7).

### 3.4. Synthesis of 4,7-Dibromobenzo[c][1,2,5]thiadiazole-5,6-diamine (BT–2NH_2_)

BT–2NH_2_ was synthesized via the reduction reaction of BT–2NO_2_ (500 mg, 1.3 mmol) in 20 mL of acetic solution in the presence of Fe (0.875 mg, 15.67 mmol) at room temperature and acetic acid (10 mL). The mixture solution was added into ice water to obtain yellow powder (400 mg, 80%). FTIR (Figure S8): 3323, 3290 cm^−1^ (NH_2_ group). ^1^H NMR (Figure S9): 4.52 ppm (4H, 2NH_2_). (+) ESI-MS (Figure S10): 322.90. T_d10_ = 228 °C (Figure S11).

### 3.5. Synthesis of 4,8-Dibromobenzo(1,2-c;4,5-c′)bis(1,2,5)thiadiazole (BBT–Br_2_)

In 100 mL of three necks under N_2_ environment, BT–2NH_2_ (500 mg, 1.54 mmol) was degassed twice a time under vacuum; then, DCM (10 mL), triethylamine (860 µL, 6.17 mmol), and thionyl chloride (220 µL, 3.1 mmol) were added at 0 °C. Afterward, the mixture was stirred and heated for 12 h at 50 °C, and DCM was removed using rotary evaporation; then, the mixture was added to 3N HCl and stirred for 30 min. Finally, it was filtered and washed using MeOH and reprecipitated by DCM and MeOH; thus, a dark red powder was obtained (368 mg, 68%). FTIR (Figure S12): 3050, 1611 cm^−1^ (NH_2_ group). ^1^H NMR (Figure S13): no signal observed. (+) ESI-MS (Figure S14): 353. T_d10_ (thermal decomposition temperatures at 10% weight loss) = 262 °C (Figure S15).

### 3.6. Synthesis of Tris(4-((trimethylsilyl)ethynyl)phenyl)amine (TPA–TMS)

Pd(PPh_3_)_4_ (0.150 mmol), PPh_3_ (0.470 mmol), CuI (0.310 mmol), and TPA–Br_3_ (2.07 mmol) were mixed in THF (50 mL). Trimethylsilyl acetylene (1.42 g, 15.5 mmol) was added, and the mixture was refluxed for 3 days. After cooling, the contents inside the flask were endowed for cooling and then filtered off and extracted with DCM (50 mL); then, the solvent was evaporated, and the pure TPA–TMS as a yellow solid (900 mg, 90%) was obtained after running column chromatography (Scheme S1). FTIR (Figure S16a) 3035, 2109 (C≡C stretching) cm^−1^. ^1^H NMR (Figure S17a): 7.34 (s, 6H), 6.97 (s, 6H), 0.257 (s, 27H, CH_3_) ppm. ^13^C NMR (Figure S17c): 148.14, 134.03, 124.83, 118.49, 105.83, 93.83, 0.09 ppm.

### 3.7. Synthesis of Tris(4-ethynylphenyl)amine (TPA-T)

TPA–TMS (0.520 g, 0.970 mmol) and K_2_CO_3_ (1.00 g, 7.24 mmol) were stirred in MeOH (25 mL) at 27 °C for 1 d. The solvent was evaporated and added to H_2_O (100 mL) to yield TPA-T (450 mg, 87%, Scheme S1). FTIR (Figure S16b): 3276 (≡C–H), 2109 (C≡C stretching) cm^−1^. ^1^H NMR (Figure S17b): 7.39 (s, 6H), 7.03 (s, 6H), 3.04 (s, 3H) ppm. ^13^C NMR (Figure S17d): 148.48, 134.03, 124.15, 117.8, 83.28 ppm.

### 3.8. Synthesis of BBT–CMP Materials

In the Schlenk storage tube, 0.1 g of TPA–Br_3_ or 0.1 g of Py–Br_4_ or 0.1 g of TPE–Br_4_ was dissolved in the presence of CuI, PPh_3_, Pd(PPh_3_)_4,_ and BBT–Br_2_ in a mixture of DMF and Et_3_N. Further, the tube was stirred and heated for 72 h at 100 °C. The obtained product was washed with different organic solvents such as DMF, THF, and acetone. The product was dried at 70 °C to obtain brown powder for TPA–BBT–CMP (0.08 g, 80%, Scheme 2e); reddish-brown powder for Py–BBT–CMP (0.09 g, 90%, Scheme 2f); orange powder for TPE–BBT–CMP (0.07 g, 70%, Scheme 2g).

## 4. Characterization

FTIR spectra were recorded using a Bruker Tensor 27 FTIR spectrophotometer with a resolution of 4 cm^−1^ through the KBr disk method. ^13^C Nuclear magnetic resonance (NMR) spectra were recorded using an INOVA 500 instrument with DMSO-*d*_6_ as the solvent and TMS as the external standard; chemical shifts are reported in parts per million (ppm). The thermal stabilities of the samples were examined under a N_2_ atmosphere using a TG Q-50 thermogravimetric analyzer; each cured sample (ca. 5 mg) was placed in a Pt cell and heated at a rate of 20 °C min^−1^ from 100 to 800 °C under a N_2_ flow rate of 60 mL min^−1^. Wide-angle X-ray diffraction (WAXD) patterns were measured using the wiggler beamline BL17A1 of the National Synchrotron Radiation Research Center (NSRRC), Taiwan; a triangular bent Si (111) single crystal was used to give a monochromated beam having a wavelength (λ) of 1.33 Å. The morphologies of the polymer samples were examined using field emission scanning electron microscopy (FE-SEM; JEOL JSM7610F) and transmission electron microscopy (TEM), using a JEOL-2100 instrument operated at an accelerating voltage of 200 kV. The Brunauer–Emmett–Teller (BET) surface areas and porosities of the samples (ca. 40–100 mg) were measured using BEL MasterTM/BEL simTM (v. 3.0.0). N_2_ adsorption and desorption isotherms were generated through incremental exposure to ultrahigh purity N_2_ (up to ca. 1 atm) in a liquid N_2_ (77 K) bath. Surface parameters were calculated using the BET adsorption models in the instrument’s software. The pore sizes of the prepared samples were determined using nonlocal density functional theory (NLDFT).

## 5. Conclusions

In conclusion, three types of CMPs (TPA–BBT, Py–BBT, and TPE–BBT–CMPs) based on BBT unit were successfully synthesized via Sonogashira coupling’s reaction, for application as electrode materials in energy storage systems. Several techniques were employed to characterize the porosity, thermal stability, and morphologies of the new BBT–CMP properties such as BET, TGA, SEM, and TEM. More interestingly, TPA–BBT–CMP, Py–BBT–CMP, and TPE–BBT–CMP electrodes featured high specific capacitance values of 220, 228, and 214 F g^−1^, respectively, at a current of 0.5 A g^−1^, and they could retain their durability up to after 2000 cycles at a current of 10 A g^−1^, due to their highly delocalized LUMO distribution and highly delocalized LUMO distribution and small bandgaps (based on DFT and MESP analyses). The unique properties of BBT–CMPs, as shown above, make them ideal candidates for energy storage and other potential applications.

## Figures and Tables

**Table 1:** A summary of the TGA and porosity properties of BBT–CMPs materials in this study.

Material	T_d10_ (°C)	Char Yield (%)	Surface Area (m^2^ g^−1^)	Pore Size (nm)
TPA–BBT–CMP	250	55	35.6	2.7
Py–BBT–CMP	244	48	67	1.3
TPE–BBT–CMP	347	67	410	1.8

## Data Availability

The data presented in this study are available in the article and supplementary material.

## Supplementary Materials

The following supporting information can be downloaded at: https://www.mdpi.com/article/10.3390/molecules27062025/s1, Scheme S1: Syntheses of (a) TPA–TMS and (b) TPA-T, Figure S1: FTIR spectrum of BT–Br_2_, Figure S2: ^1^H-NMR spectrum of BT–Br_2_, Figure S3: ^13^C-NMR spectrum of BT–Br_2_, Figure S4: FTIR spectrum of BT–2NO_2_, Figure S5: ^1^H-NMR spectrum of BT–2NO_2_, Figure S6: (+) ESI-MS spectrum of BT–2NO_2_, Figure S7: TGA profile of BT–2NO_2_, Figure S8: ^1^H-NMR spectrum of BT–2NH_2_, Figure S9: ^1^H-NMR spectrum of BT–2NH_2_, Figure S10” (+) ESI-MS spectrum of BT–2NH_2_, Figure S11: TGA profile of BT–2NH_2_, Figure S12: ^1^H-NMR spectrum of BBT–Br_2_, Figure S13: ^1^H-NMR spectrum of BBT–Br_2_, Figure S14: (+) ESI-MS spectrum of BBT–Br_2_, Figure S15: TGA profile of BBT–Br_2_, Figure S16: FTIR spectra of (a) TPA–TMS and (b) TPA-T, Figure S17: (a,b) ^1^H and (c,d) ^13^C NMR spectra of (a,c) TPA–TMS and (b,d) TPA-T, Figure S18: Stability performance of (a) TPA–BBT–CMP, (b) Py–BBT–CMP, and (c) TPE–BBT–CMP after 2000 cycles, Figure S19: Energy level profiles of (a) BBT, (b) TPA, (c) Py, and (d) TPE compounds, Figure S20: The molecular electrostatic potential of (a) BBT, (b) TPA, (c) Py and (d) TPE, Table S1: Summarized the thermal stability of BT–2NO_2_, BT–2NH_2_, BBT–Br_2,_ TPA–BBT–CMP, Py–BBT–CMP, and TPE–BBT–CMP, Table S2: Comparison between the specific surface area/specific capacitance of BBT–CMP samples with those of previously reported materials for supercapacitor application [2,10,12,13,19,33,73,74,75,76].

## Sample Availability

Samples of the compounds are not available from the authors.
