# Supplementary material for: Ultrastable Conjugated Microporous Polymers Containing Benzobisthiadiazole and Pyrene Building Blocks for Energy Storage Applications
Source: Molecules. 2022 Mar 21;27(6):2025. doi: 10.3390/molecules27062025 (PMC8952824; doi:10.3390/molecules27062025)
Supplement: Supplementary file 1 [file molecules-27-02025-s001.zip › molecules-1642910-supplementary.pdf]

Supporting Information for

Ultrastable Conjugated Microporous Polymers Containing Benzobisthiadiazole and Pyrene Building Blocks for Energy Storage Applications

Mohamed Gamal Mohamed [1,2,†], Tharwat Hassan Mansoure [2,†], Maha Mohamed Samy [1,2], Yasuno Takashi [1], Ahmed A. K. Mohammed [2], Tansir Ahamad [3], Saad M. Alshehri [3], Jeonghun Kim [4], Babasaheb M. Matsagar [5], Kevin C. -W. Wu [5,*], and Shiao-Wei Kuo [1,6,*]

[1] Center of Crystal Research, Department of Materials and Optoelectronic Science, National Sun Yat-Sen University, Kaohsiung 804, Taiwan; mgamal.eldin12@aun.edu.eg (M.G.M.); d083100006@nsysu.edu.tw (M.M.S.); yasunou21@hotmail.co.jp (Y.T.)
[2] Department of Chemistry, Faculty of Science, Assiut University, Assiut 71516, Egypt; tharout.mansour@science.au.edu.eg (T.H.M.); theoahmedkamel@gmail.com (A.A.K.M.)
[3] Department of Chemistry, College of Science, King Saud University, Riyadh 11362, Saudi Arabia; tahamed@ksu.edu.sa (T.A.); alshehri@ksu.edu.sa (S.M.A.)
[4] Department of Chemical and Biomolecular Engineering, Yonsei University, 50 Yonsei-ro, Seodaemun-gu, Seoul 03722, Korea; jeonghun.kim@uq.edu.au
[5] Department of Chemical Engineering, National Taiwan University, No. 1, Sec. 4, Roosevelt Road, Taipei 10617, Taiwan; matsagar03@ntu.edu.tw
[6] Department of Medicinal and Applied Chemistry, Kaohsiung Medical University, Kaohsiung 807, Taiwan
* Correspondence: kevinwu@ntu.edu.tw (K.C.-W.W.); kuosw@faculty.nsysu.edu.tw (S.-W.K.)
† These authors contributed equally to this work.

Materials

Tetraphenylethene (TPE), 1,1,2,2-tetrakis(4-bromophenyl)ethene (TPE-Br_4), tri(p-bromophenyl)triphenylamine (TBA-Br_3), 1,3,6,8-tetrabromopyrene (Py-Br_4), Py-T, and TPE-T were synthesized according to previously reported procedures [2,19,33,73–75].

Electrochemical Analysis

Working Electrode Cleaning: Prior to use, the glassy carbon electrode (GCE) was polished several times with 0.05-μm alumina powder, washed with EtOH after each polishing step, cleaned through sonication (5 min) in a water bath, washed with EtOH, and then dried in air.

Electrochemical Characterization: The electrochemical experiments were performed in a three-electrode cell using an Autolab potentiostat (PGSTAT204) and 1 M KOH as the aqueous electrolyte. The GCE was used as the working electrode (diameter: 5.61 mm; 0.2475 cm^2); a Pt wire was used

as the counter electrode; Hg/HgO (RE-1B, BAS) was the reference electrode. All reported potentials refer to the Hg/HgO potential. A slurry was prepared by dispersing the sample (45 wt. %), carbon black (45 wt. %), and Nafion (10 wt. %) in a mixture of (EtOH/ H_2O) (200 μL: 800 μL) and then sonicating for 1 h. A portion of this slurry (10 μL) was pipetted onto the tip of the electrode, which was then dried in air for 30 min prior to use. The electrochemical performance was studied through CV at various sweep rates (5–200 mV s^{-1}) and through the GCD method in the potential range from 0.10 to –0.90 V (vs. Hg/HgO) at various current densities (0.5–20 A g^{-1}) in 1 M KOH as the aqueous electrolyte solution.

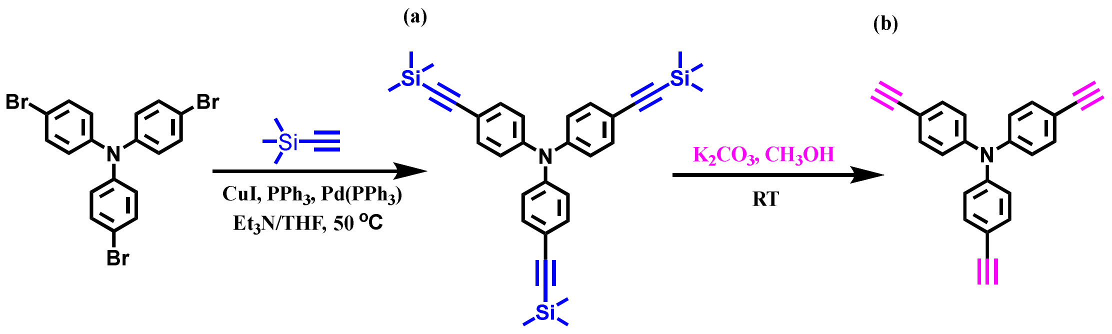

Scheme S1. Syntheses of (a) TPA-TMS and (b) TPA-T.

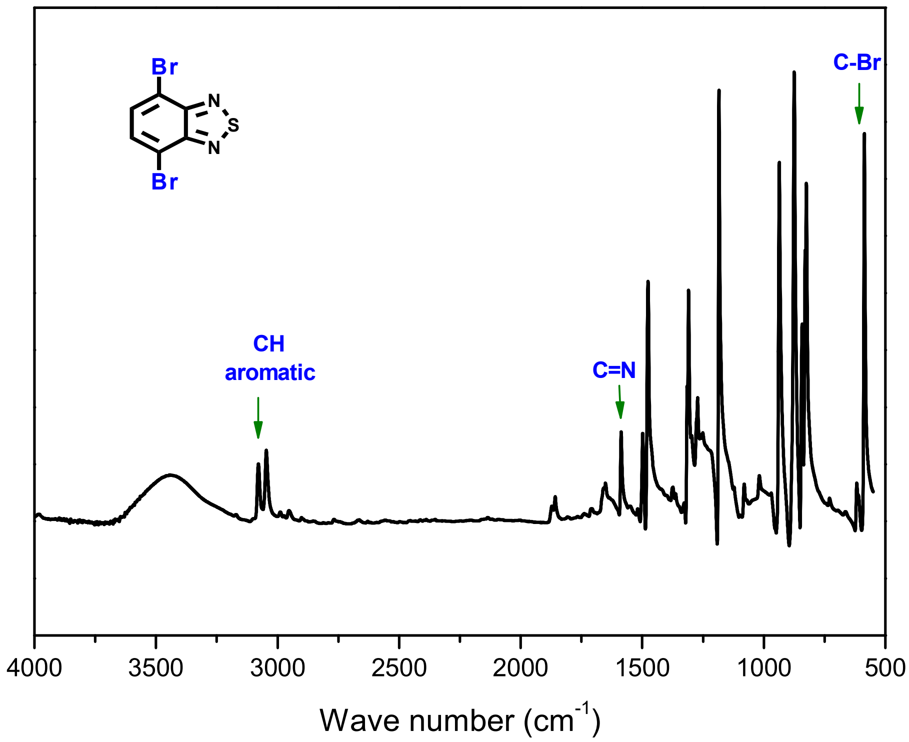

Figure S1. FTIR spectrum of BT-Br_2.

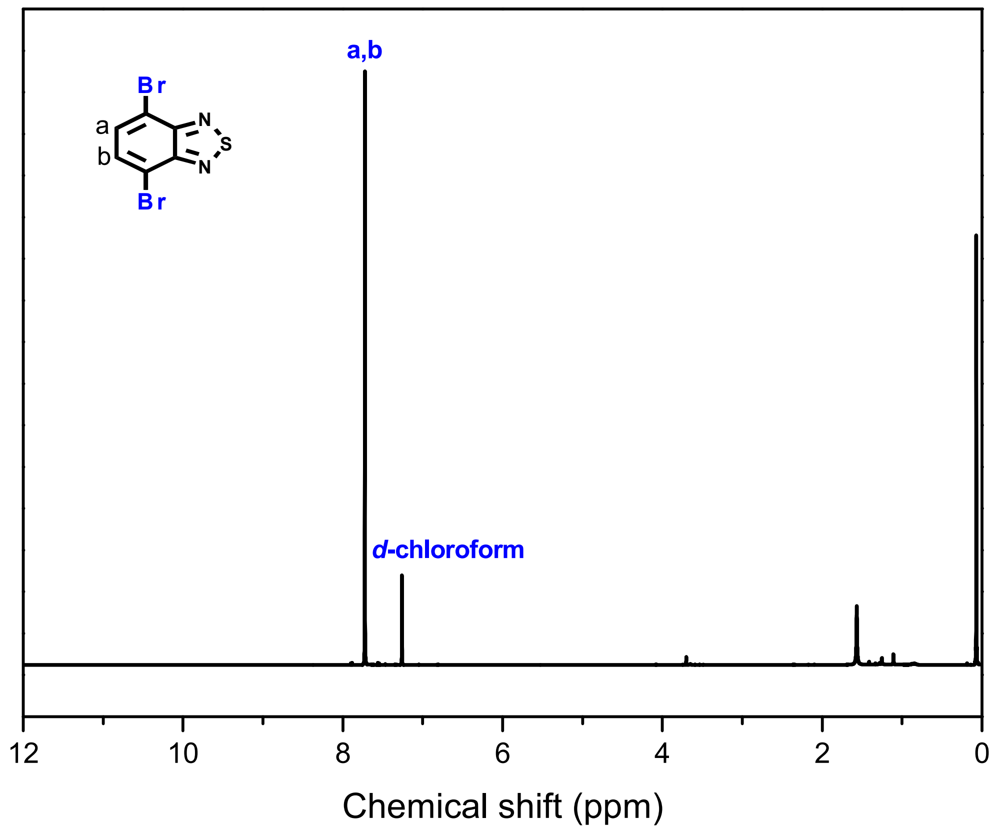

Figure S2. ^{1}H-NMR spectrum of BT-Br_2.

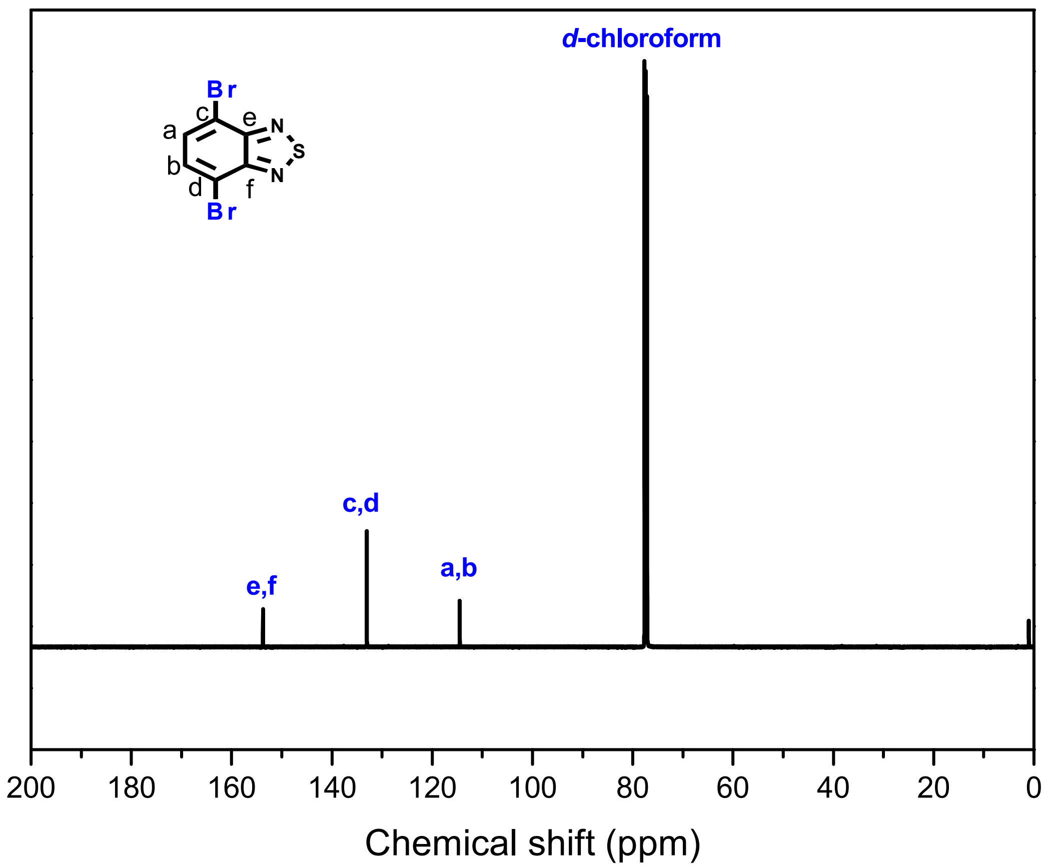

Figure S3. ^{13}C-NMR spectrum of BT-Br_2.

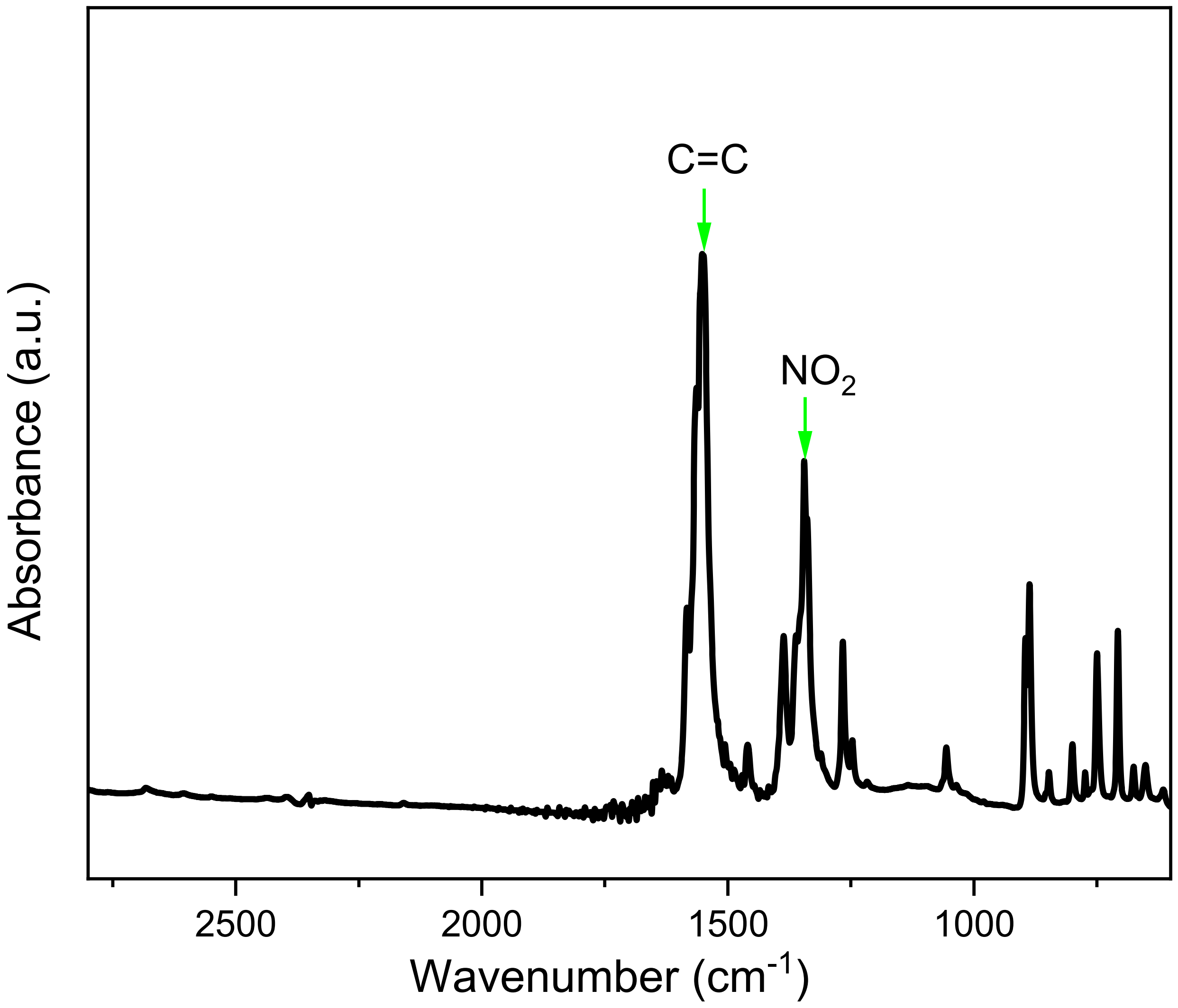

Figure S4. FTIR spectrum of BT-2NO_2.

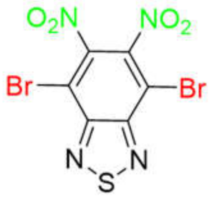

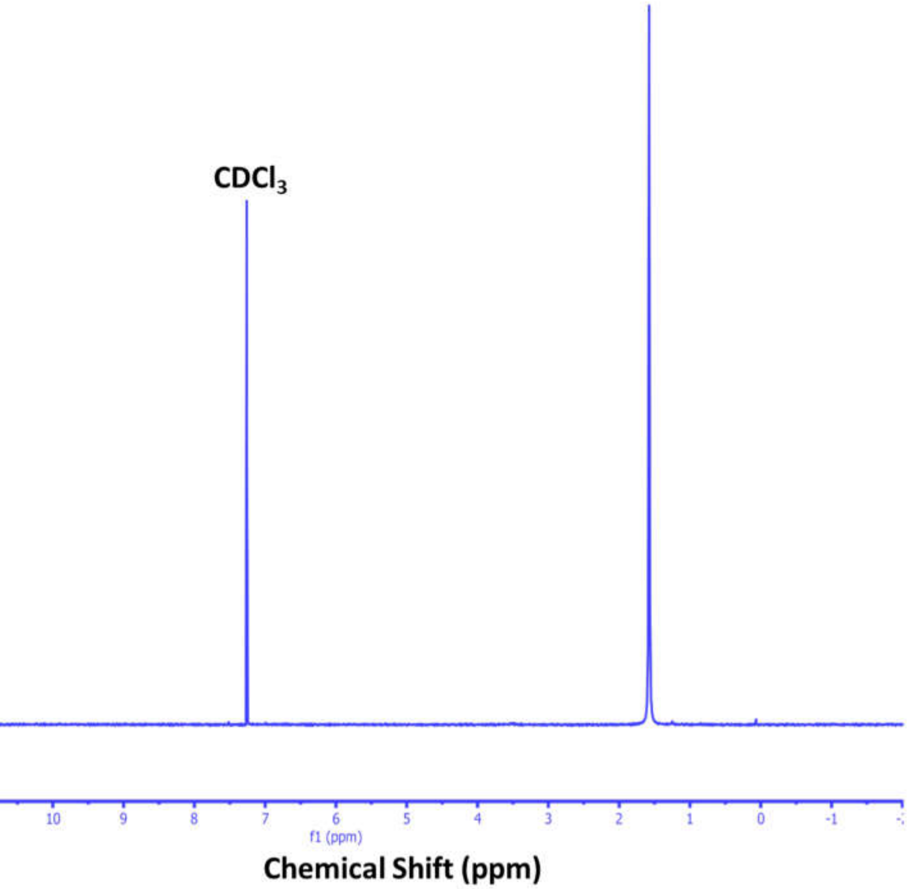

Figure S5. ^{1}H-NMR spectrum of BT-$2NO_2$.

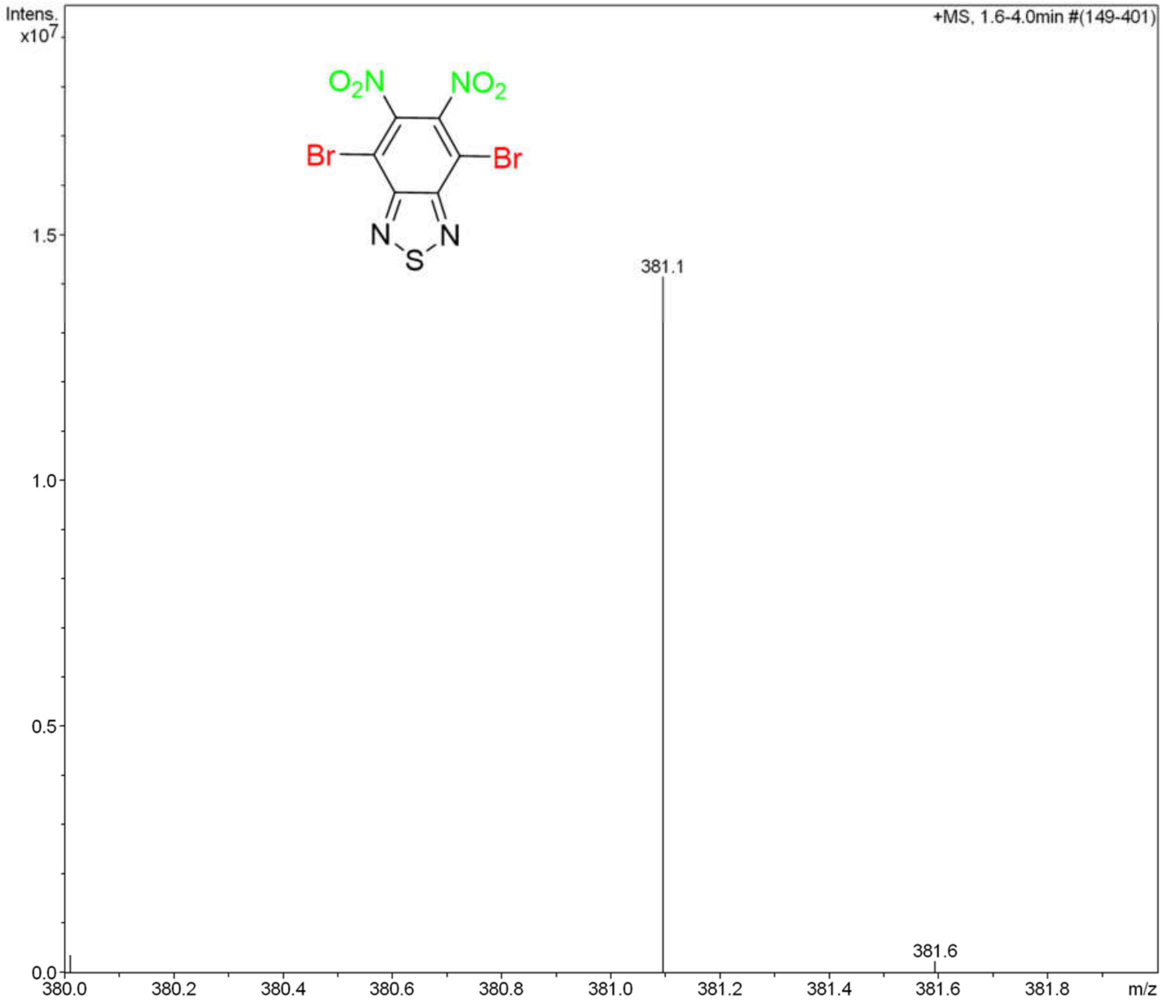

Figure S6. (+) ESI-MS spectrum of BT-2NO_2.

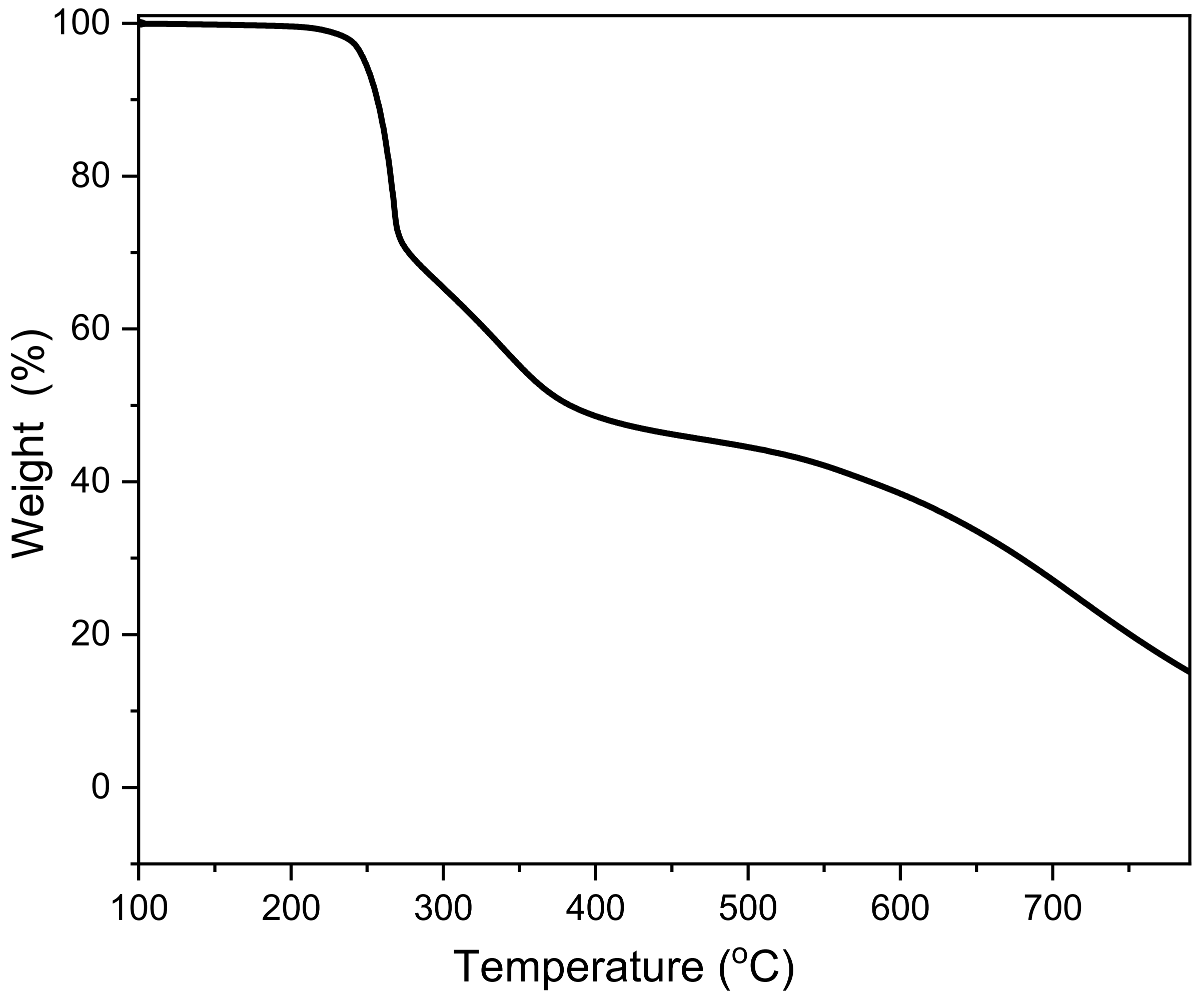

Figure S7. TGA profile of BT-$2NO_2$.

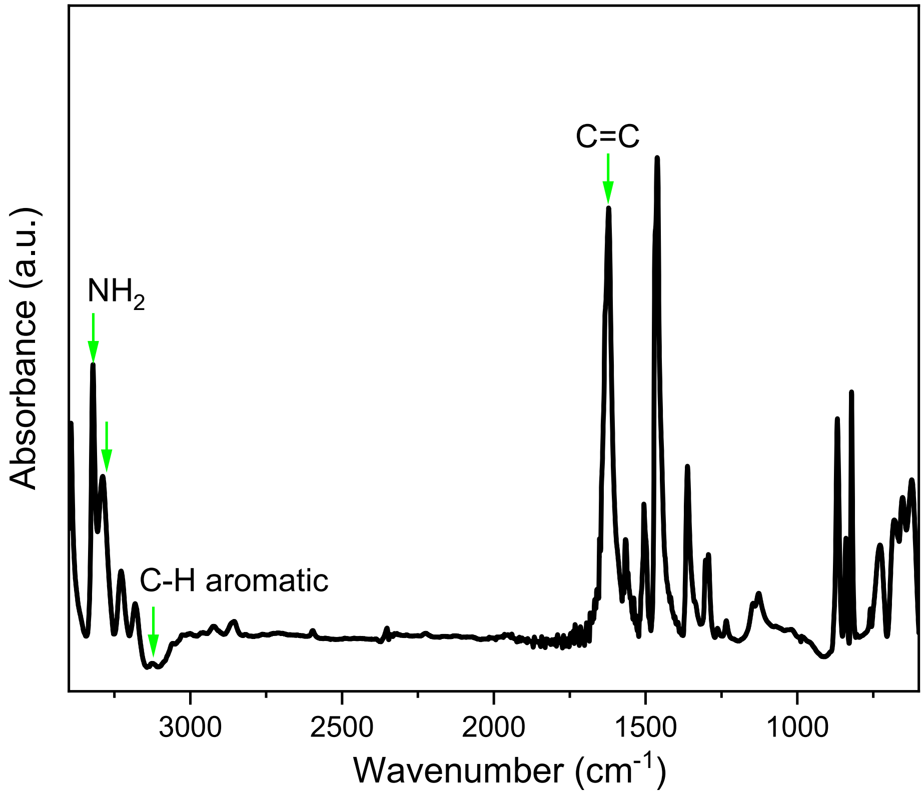

Figure S8. ^{1}H-NMR spectrum of BT-2NH$_2$.

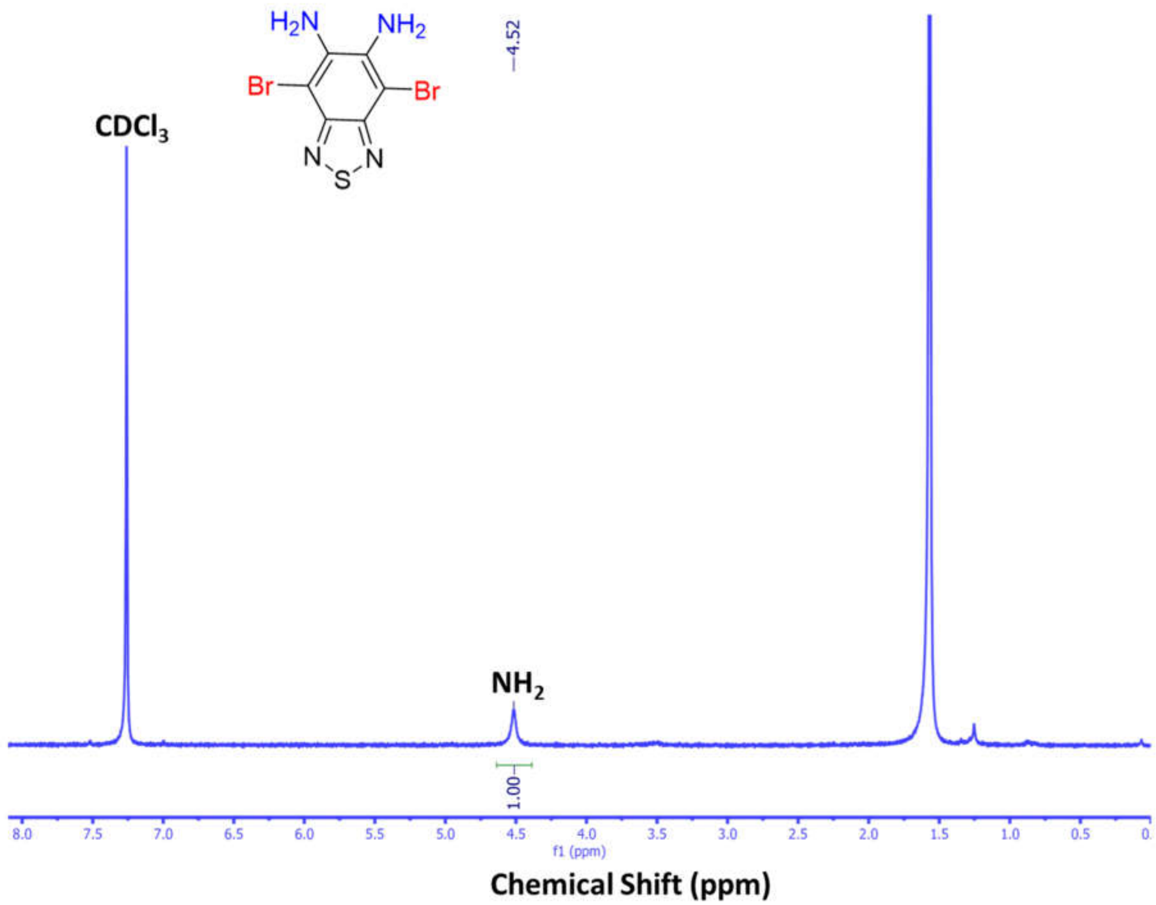

Figure S9. ^{1}H-NMR spectrum of BT-2NH_2.

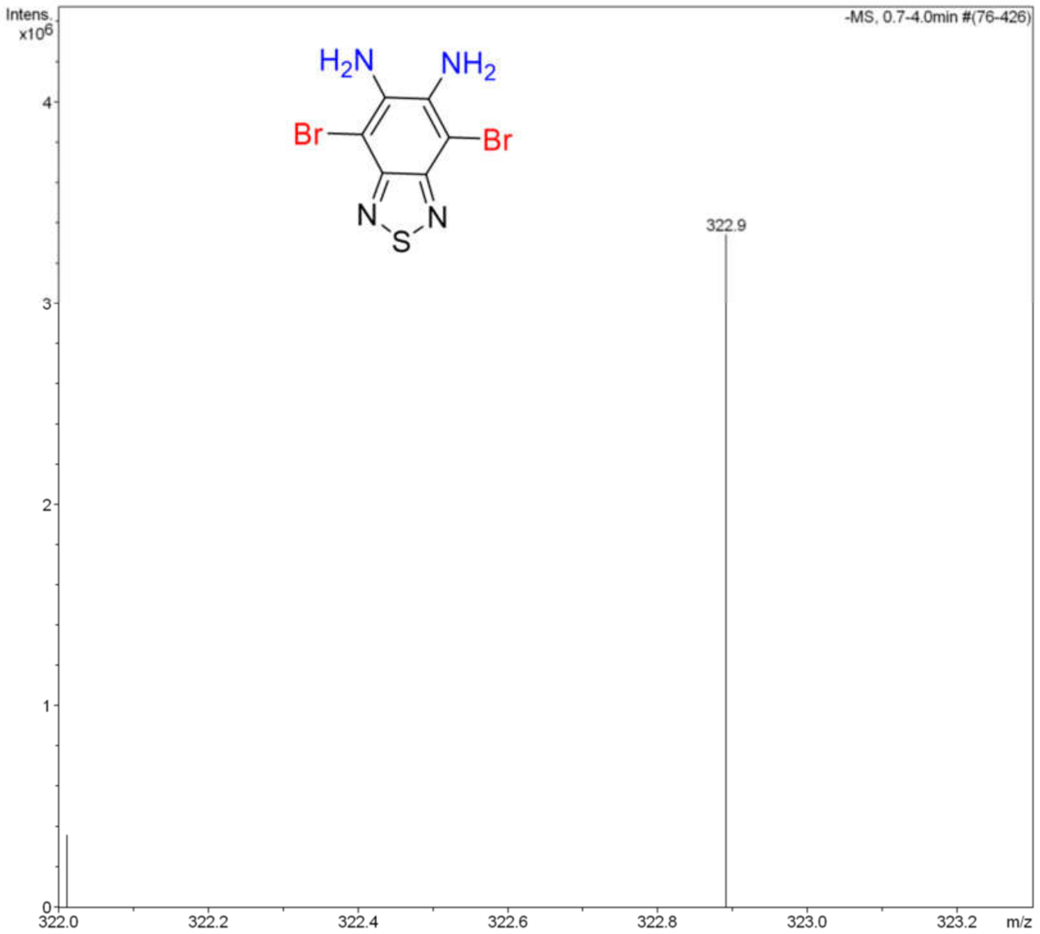

Figure S10. (-) ESI-MS spectrum of BT-$2NH_2$.

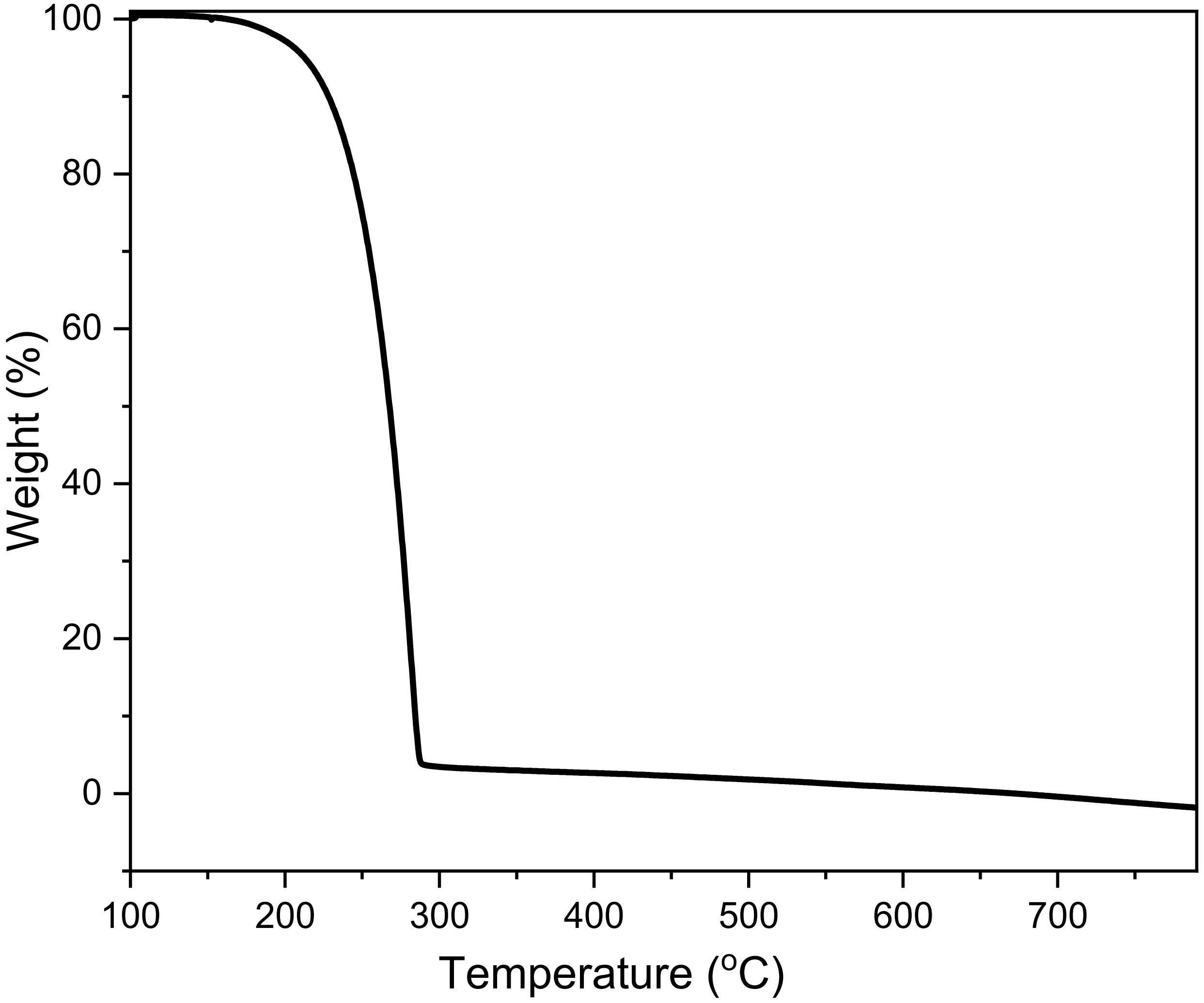

Figure S11. TGA profile of BT-$2NH_2$.

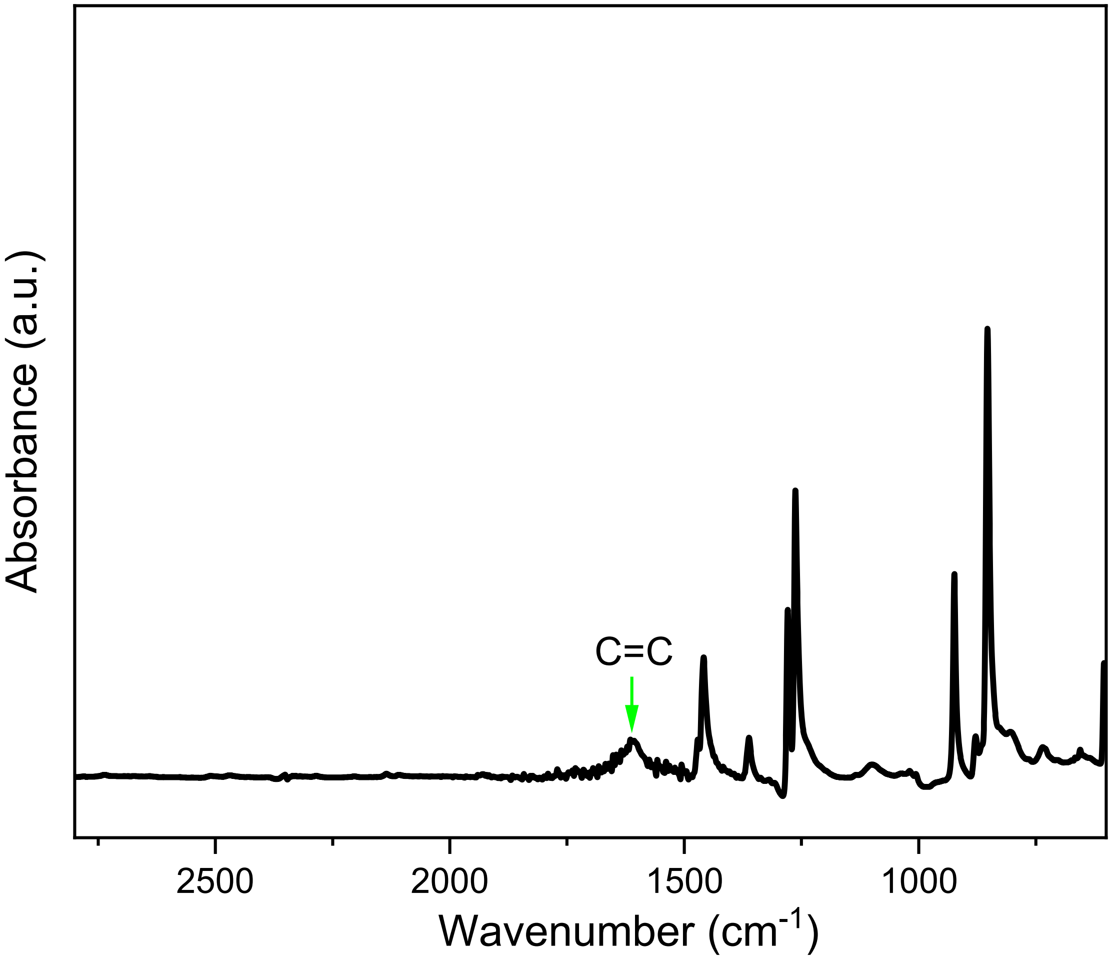

Figure S12. ^{1}H-NMR spectrum of BBT-Br_2.

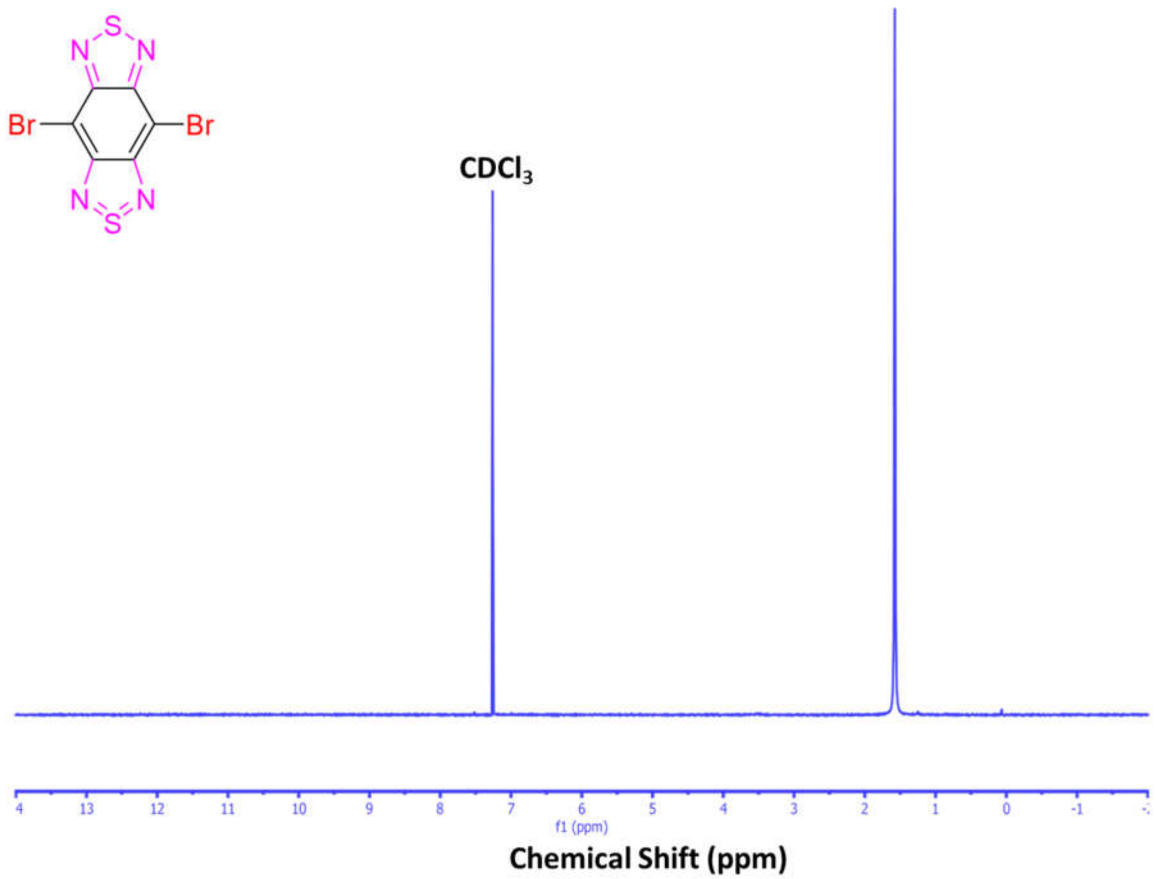

Figure S13. ^{1}H-NMR spectrum of BBT-Br_2.

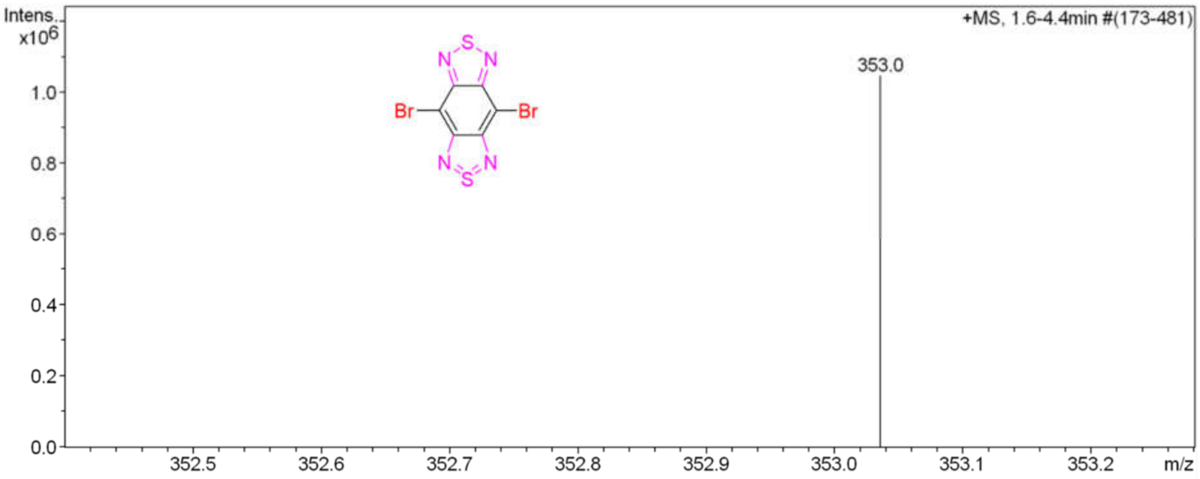

Figure S14. (+) ESI-MS spectrum of BBT-Br_2.

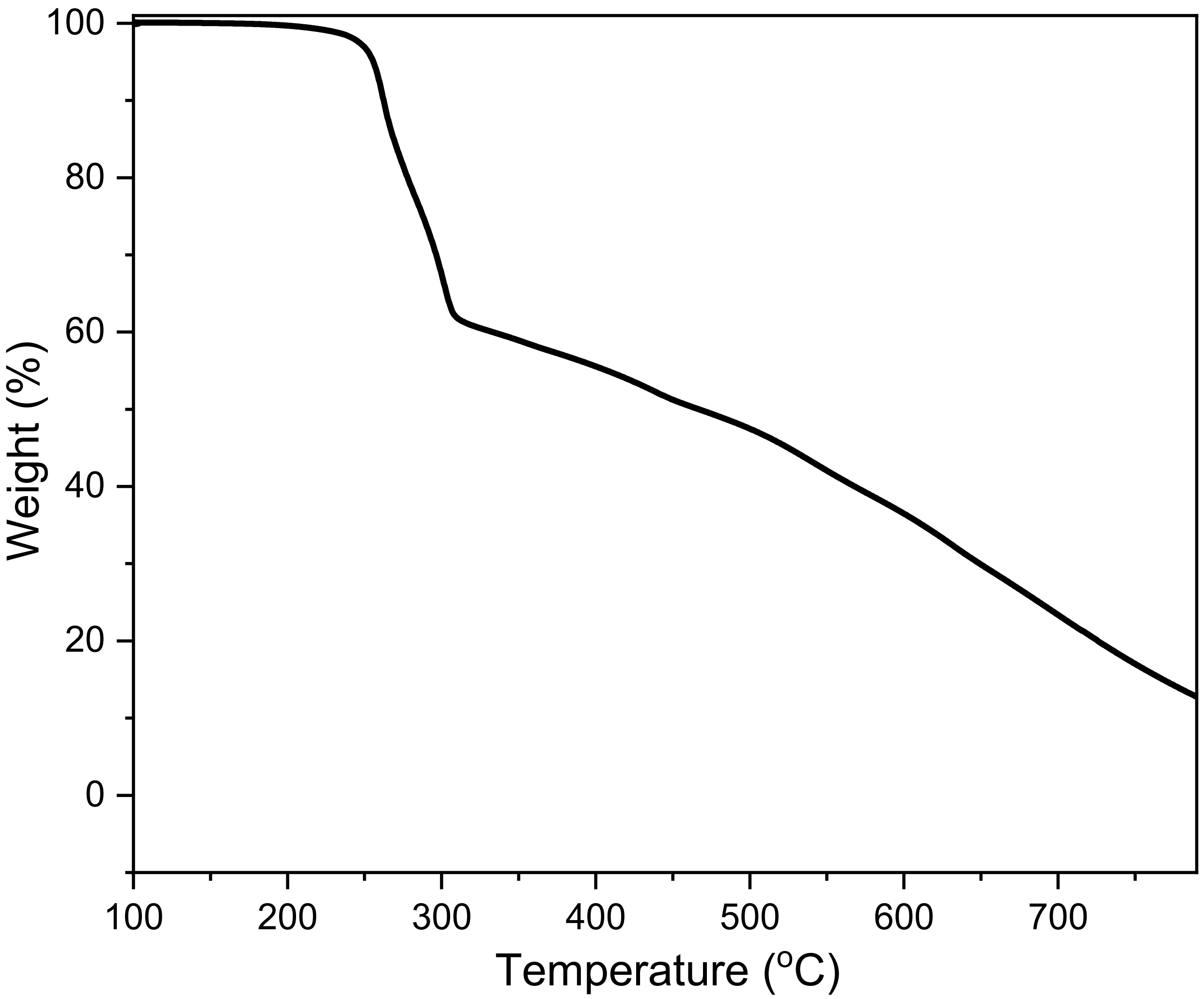

Figure S15. TGA profile of BBT-Br_2.

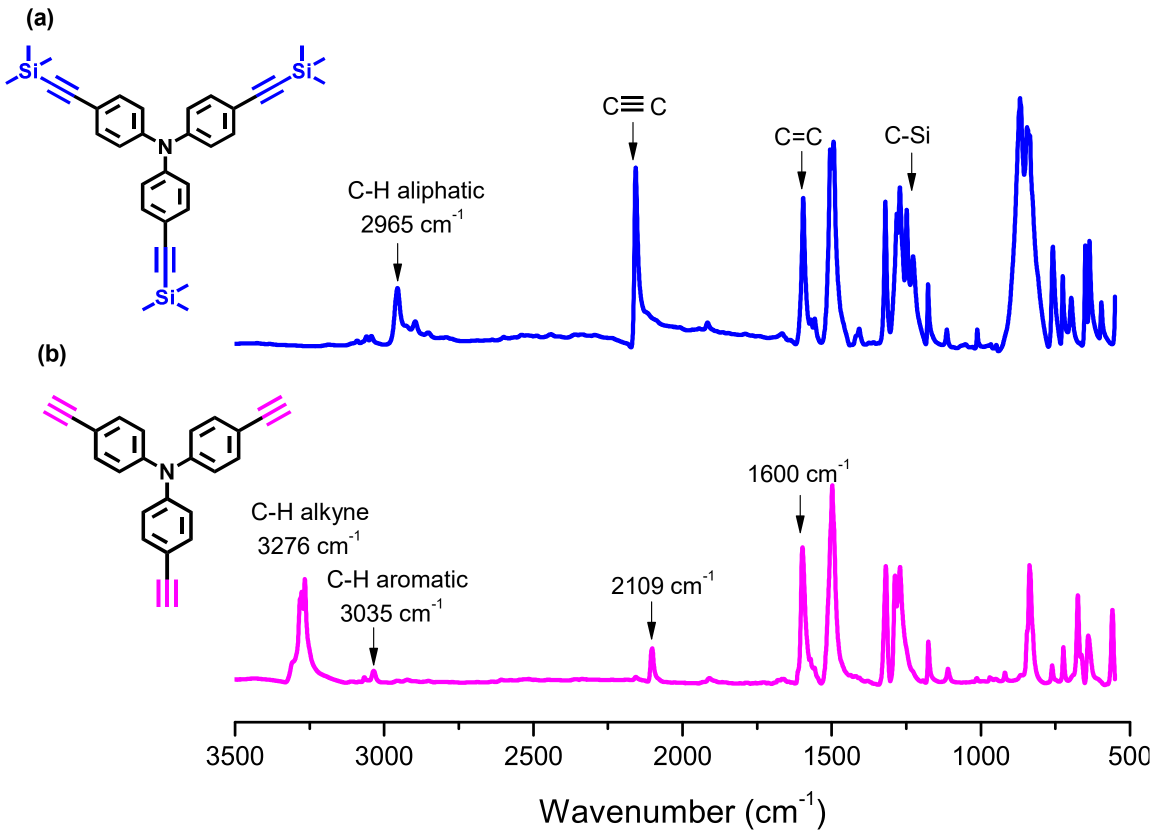

Figure S16. FTIR spectra of (a) TPA-TMS and (b) TPA-T.

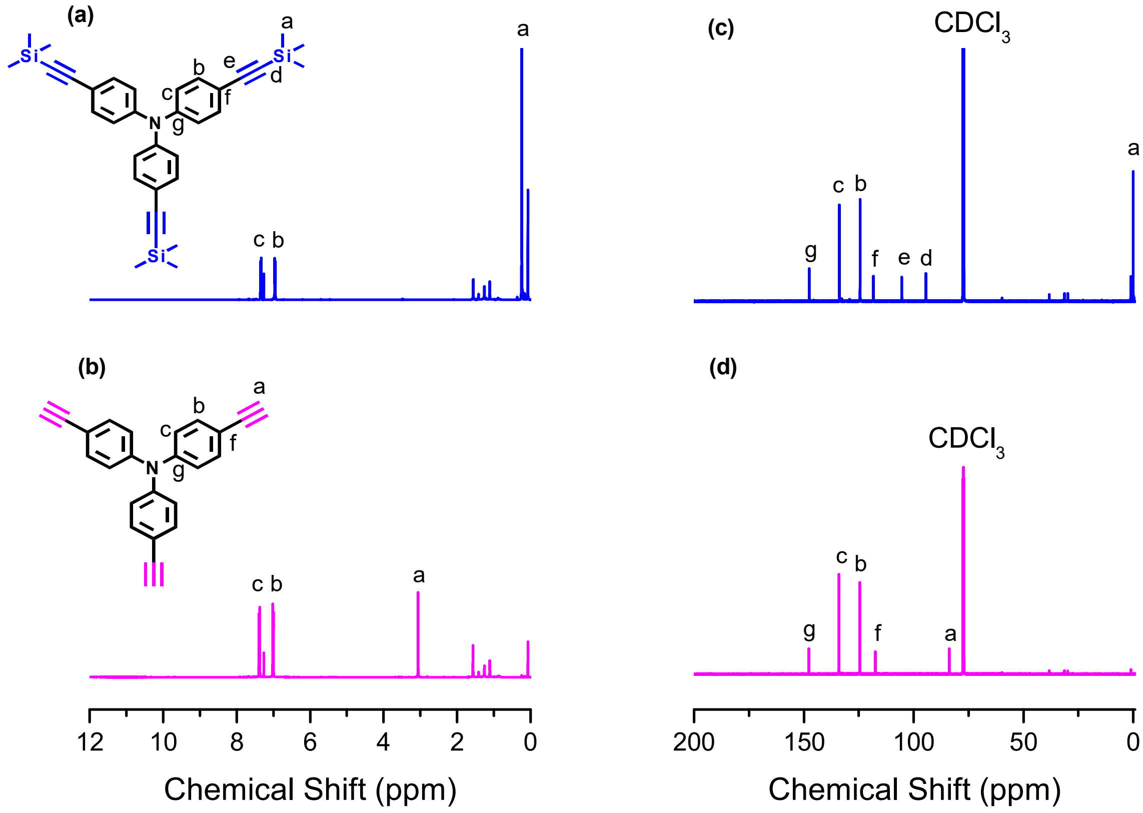

Figure S17. (a, b) ^{1}H and (c, d) ^{13}C NMR spectra of (a, c) TPA-TMS and (b, d) TPA-T.

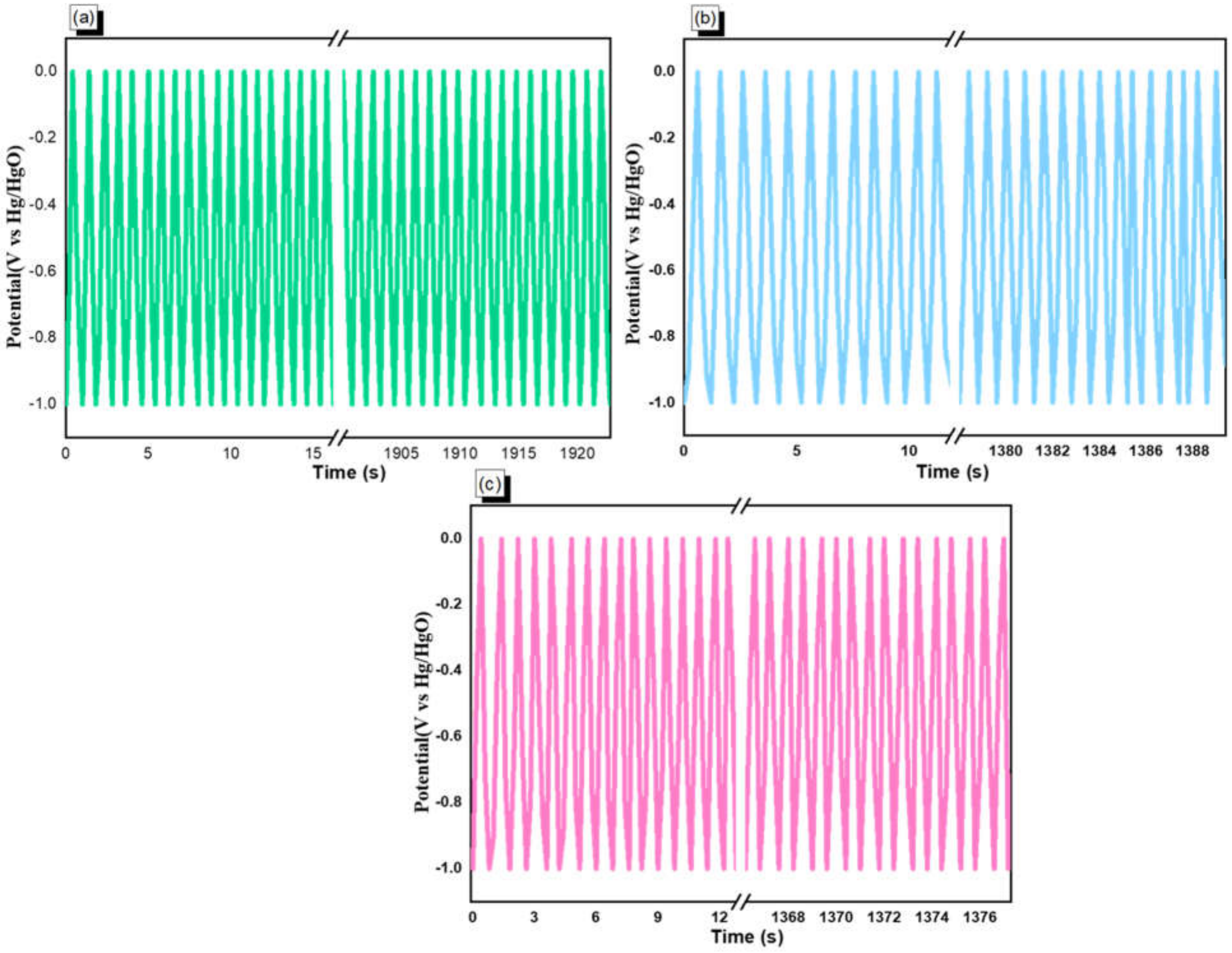

Figure S18. Stability performance of (a) TPA-BBT-CMP, (b) Py-BBT-CMP and (c) TPE-BBT-CMP after 2000 cycles.

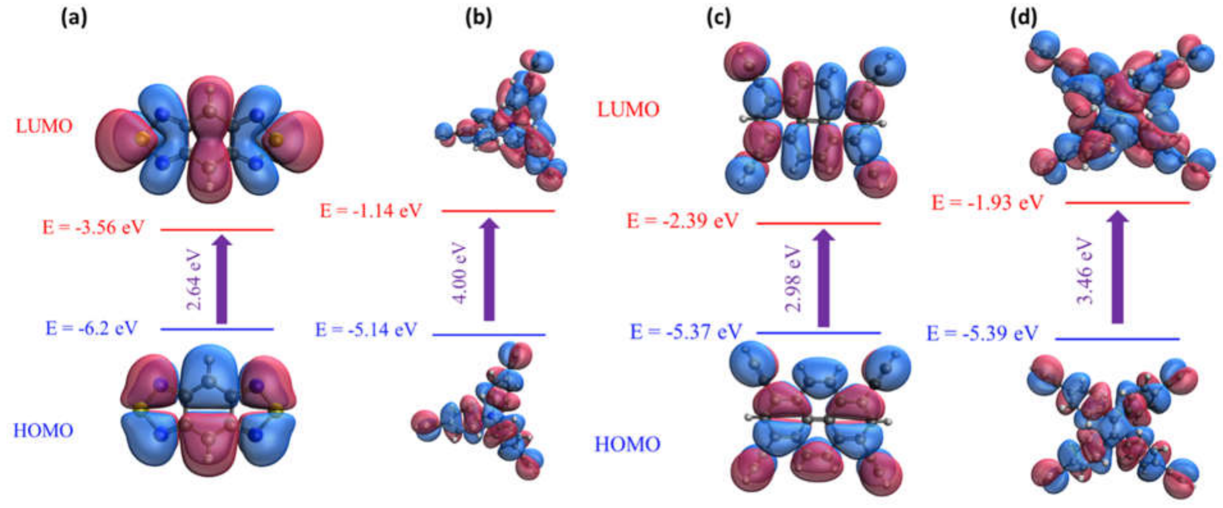

Figure S19. Energy level profiles of (a) BBT, (b) TPA, (c) Py and (d) TPE compounds.

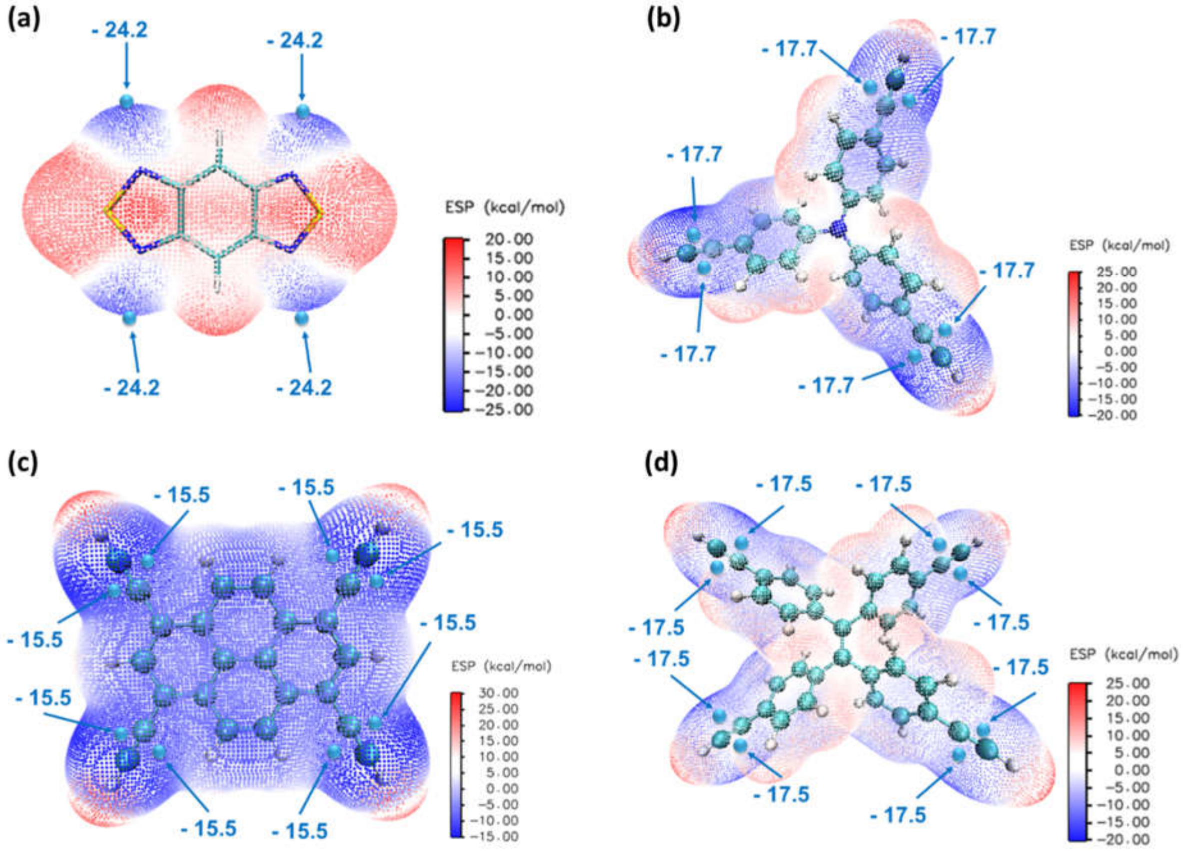

Figure S20. the molecular electrostatic potential of (a) BBT, (b) TPA, (c) Py and (d) TPE.

Table S1. Summarized the thermal stability of BT-2NO_2, BT-2NH_2, BBT-Br_2, TPA-BBT-CMP, Py-BBT-CMP and TPE-BBT-CMP.

Sample	T_{d5} (°C)	T_{d10} (°C)	Char Yield (%)
BT-2NO_2	248	256	15
BT-2NH_2	212	228	0
BBT-Br_2	255	262	13
TPA-BBT-CMP	221	250	55
Py-BBT-CMP	198	244	48
TPE-BBT-CMP	272	347	67

Table S2. Comparison between the specific surface area/specific capacitance of BBT-CMPs samples with those of previously reported materials for supercapacitor application.

Electrode	S_{BET} ($m^2\ g^{-1}$)	Capacitance	Ref.
TPA-BBT-CMP	35.6	220 F g^{-1} at 0.5 A g^{-1}	This work
Py-BBT-CMP	67	228 F g^{-1} at 0.5 A g^{-1}	This work
TPE-BBT-CMP	410	214 F g^{-1} at 0.5 A g^{-1}	This work
Py-FFC-CMP	50	5.07 F g^{-1} at 0.5 A g^{-1}	[12]
TPE-FFC-CMP	8	4.8 F g^{-1} at 0.5 A g^{-1}	[12]
TBN-Py-CMP	473	31 F g^{-1} at 0.5 A g^{-1}	[19]
TBN-TPE-CMP	1150	18.45 F g^{-1} at 0.5 A g^{-1}	[19]
TBN-Car-CMP	762	18.90 F g^{-1} at 0.5 A g^{-1}	[19]
TBN-Py-CMP/SWCNT	-	430 F g^{-1} at 0.5 A g^{-1}	[19]
TBN-TPE-CMP/SWCNT	-	156 F g^{-1} at 0.5 A g^{-1}	[19]
TBN-Car-CMP/SWCNT	-	53 F g^{-1} at 0.5 A g^{-1}	[19]
poly(TPEP-BZ)/SWCNT-1	-	61 F g^{-1} at 0.5 A g^{-1}	[13]
poly(TPEP-BZ)/SWCNT-5	-	84 F g^{-1} at 0.5 A g^{-1}	[13]
TPA-COF-1	714	51.3 F g^{-1} at 0.2 A g^{-1}	[76]
TPA-COF-2	478	14.4 F g^{-1} at 0.2 A g^{-1}	[76]
TPA-COF-3	557	5.1 F g^{-1} at 0.2 A g^{-1}	[76]
TPT-COF-4	1132	2.4 F g^{-1} at 0.2 A g^{-1}	[76]
TPT-COF-5	1747	0.34 F g^{-1} at 0.2 A g^{-1}	[76]
TPT-COF-6	1535	0.24 F g^{-1} at 0.2 A g^{-1}	[76]
POSS-A-POIP	426	152.5 F g^{-1} at 0.5 A g^{-1}	[10]
POSS-F-POIP	452	36.5 F g^{-1} at 0.5 A g^{-1}	[10]
TPE-HPP	922	67 F g^{-1} at 0.5 A g^{-1}	[73]
DPT-HPP	1230	110.5 F g^{-1} at 0.5 A g^{-1}	[73]
